# Comparative Analysis of Aggregation of β- and γ-Synucleins in Vertebrates

**DOI:** 10.3390/biom15091231

**Published:** 2025-08-26

**Authors:** Maria Carmela Bonaccorsi di Patti, Martina Meoni, Mattia Toni

**Affiliations:** 1 Department of Biochemical Science “A. Rossi Fanelli”, Sapienza University of Rome, 00185 Rome, Italy; mariacarmela.bonaccorsi@uniroma1.it (M.C.B.d.P.); meoni.1886166@studenti.uniroma1.it (M.M.); 2 Department of Biology and Biotechnologies “Charles Darwin”, Sapienza University, 00185 Rome, Italy

**Keywords:** β-synuclein, γ-synuclein, copper ions, thioflavin T fluorescence, circular dichroism, vertebrates

## Abstract

This study explores the structural transitions and aggregation behaviour of recombinant β- and γ-synucleins from five vertebrate species—*Cyprinus carpio*, *Danio rerio*, *Xenopus laevis*, *Anolis carolinensis,* and *Homo sapiens*—using thioflavin T fluorescence and circular dichroism spectroscopy, with and without copper ions. Although synucleins are well-conserved proteins among vertebrates, species-specific differences in amino acid composition and predicted secondary structures were observed, particularly within β-strand-forming regions. During a six-day incubation, human β-synuclein exhibited a time-dependent increase in β-sheet-rich structures, while non-mammalian β-synucleins showed limited variation. In contrast, γ-synucleins from all species displayed greater aggregation propensity, with variations in kinetics and magnitude. The presence of copper reduced the rate of aggregation in human β-synuclein, likely due to high-affinity metal-binding sites, whereas γ-synuclein aggregation was only mildly affected. Notably, copper enhanced late-phase aggregation in *A. carolinensis* β-synuclein. These findings suggest that sequence divergence among synuclein isoforms may underlie species-specific aggregation mechanisms and metal sensitivity. The differential aggregation behaviour observed across taxa may reflect evolutionary adaptations in synuclein function and folding propensity, with implications for understanding the molecular basis of synucleinopathies and their potential modulation by metal ions.

## 1. Introduction

Synucleins constitute a small, evolutionarily conserved family of vertebrate proteins encompassing three isoforms: α-synuclein (α-syn), β-synuclein (β-syn), and γ-synuclein (γ-syn) [1]. These proteins are characterised by notable biochemical properties, especially their conformational plasticity, and by their involvement in various neurodegenerative disorders and certain cancers.

Each isoform is encoded by a separate gene—*SNCA*, *SNCB*, and *SNCG*—located on human chromosomes 4q21.3–q22, 5q35, and 10q23, respectively [2,3,4,5,6]. α-syn and β-syn are predominantly expressed in the central nervous system [7,8,9], particularly in the presynaptic terminals of the neocortex, hippocampus, striatum, thalamus, and cerebellum [10,11]. γ-syn, initially identified in the peripheral nervous system and in breast cancer tissues [12,13], is also expressed in various brain regions, including the substantia nigra [2,14,15].

The three isoforms share substantial sequence homology, particularly in the N-terminal region. Phylogenetic evidence suggests that γ-syn is the most ancient isoform, from which α-syn and β-syn later diverged [16]. In humans, α-syn shares 62% identity (79% homology) with β-syn, and 50% identity (74% homology) with γ-syn; β-syn and γ-syn are 47% identical (66% homologous) [17]. Crucially, β-syn lacks the core NAC domain found in α-syn (residues 61–95), rendering it less prone to amyloid aggregation [18,19]. Moreover, β-syn can interact with α-syn in a chaperone-like manner, inhibiting its aggregation [20,21,22,23,24,25,26,27].

Synucleins are widely recognised as intrinsically disordered proteins [28], lacking stable secondary or tertiary structure under physiological conditions but capable of adopting α-helical or β-sheet-rich conformations [29]. This structural plasticity is critical to both their physiological function and their pathogenicity. In α-syn, α-helical states are associated with membrane binding and synaptic regulation [29], while β-sheet-rich conformers are linked to aggregation and toxicity.

α-syn and β-syn have been linked to several neurodegenerative diseases, such as Alzheimer’s disease (AD), Parkinson’s disease (PD), dementia with Lewy bodies (DLB), and multiple system atrophy (MSA) [27,30,31,32,33,34].

Numerous lines of scientific evidence support the pathological role of α-syn. Aggregated α-syn is a hallmark of synucleinopathies and is present within neuronal and glial inclusions in PD, PD dementia, DLB, and MSA [35]. In vitro, monomeric α-syn can polymerise into preformed fibrils that closely resemble those found in Lewy bodies [36]. α-syn exhibits prion-like propagation, whereby the misfolded protein serves as a template to induce further misfolding and spreads transneuronally [37].

Amino acid substitutions within the protein sequence can profoundly alter the biochemical and biophysical properties of synucleins, conferring pathological characteristics. Multiple α-syn point mutations (e.g., A30P, A53T, A53E, E46K, G51D, and H50Q) have been associated with familial forms of PD and DLB, increasing both aggregation propensity and cytotoxic potential [32,38,39,40,41]. Similarly, β-syn mutations have also been linked to neurodegenerative disease: the missense variants V70M and P123H have been identified in unrelated DLB patients [42], and these substitutions appear to increase the protein’s tendency to aggregate [42]. More recently, the M38I substitution in γ-syn has been reported in two patients with amyotrophic lateral sclerosis, where it was shown to enhance the amyloidogenic potential of γ-syn [43]. Taken together, these observations emphasise that even subtle alterations in amino acid sequence can markedly influence protein conformation, modulate aggregation kinetics, and ultimately determine disease susceptibility.

Experimental evidence further demonstrates that α-syn and β-syn can interact through the formation of heterodimers, the assembly of hetero-oligomers, and the modulation of secondary nucleation events. β-syn may act as a negative regulator of α-syn aggregation and could potentially mitigate the neuropathological alterations induced by α-syn [27,44].

γ-syn is primarily associated with oncogenic processes, and several studies have demonstrated its involvement in a range of tumour types, including breast cancer [45], endometrial cancer cell proliferation [46], oral squamous cell carcinoma [47], and biliary tract carcinoma [48]. Moreover, γ-syn has been implicated in the modulation of signal transduction pathways within nervous tissues, such as the brain and retina [27,49] and γ-syn mutation has been associated with amyotrophic lateral sclerosis [43].

The structure and aggregation propensity of synucleins can also be modulated by metal ions: Cu^2+^ promotes multimerisation [50], Mn^2+^ facilitates dityrosine crosslinking and fibrillisation [51], and Cu^+^ triggers reactive oxygen species generation and dopaminergic toxicity [52]. This phenomenon may have significant pathophysiological relevance, given that the dysregulation of metal homeostasis has been increasingly implicated in PD pathogenesis [53,54,55,56,57,58].

Given the close relationship between the structural and conformational properties of synucleins and their physiological or pathological roles, it is essential to investigate their structure in detail and to identify the conditions that drive conformational changes and promote aggregation. A diverse array of experimental approaches has been employed to elucidate synuclein structure and aggregation, including Fourier-Transform Infrared spectroscopy [59], Circular Dichroism (CD) spectroscopy [60], and solid-state Nuclear Magnetic Resonance [61]. Aggregation kinetics have been probed via Thioflavin T (ThT) fluorescence [62], Total Internal Reflection Fluorescence microscopy [63], Fluorescence Correlation Spectroscopy, Förster Resonance Energy Transfer [64], and finally Atomic Force Microscopy-Infrared spectroscopy [65].

Although α-syn remains the principal focus of neurodegeneration research, its homologues β-syn and γ-syn have received comparatively little attention, particularly in non-mammalian vertebrates. To date, only a limited number of studies have investigated these isoforms in fish [66,67,68,69,70], amphibians [71,72], reptiles [73], and birds [73,74,75], despite their evolutionary relevance and considerable structural similarity to α-syn. Research into their secondary structure and aggregation dynamics in these taxa is virtually absent. Nevertheless, the importance of elucidating the mechanisms underlying synuclein aggregation has been highlighted by recent studies on α-syn, which demonstrate how specific hydrophobic residues can govern seed-competent fibril formation [76], and how the presence of divalent and trivalent metal ions can differentially modulate aggregation dynamics [77]. Furthermore, comparative analyses have revealed that α-syn sequences from different vertebrate classes exhibit variable aggregation propensities, indicating that evolutionary divergence can markedly influence synuclein behaviour [78,79]. These observations underscore not only the pathogenic significance of aggregation mechanisms but also the potential evolutionary modulation of synuclein behaviour, thereby emphasising the necessity of extending such investigations to β-syn and γ-syn.

This study aimed to investigate the structural transitions and aggregation kinetics of β-synuclein and γ-synuclein across representative vertebrate classes, using well-established model species for which synuclein-related data are already available in the literature. To this end, we examined teleost fishes (*Cyprinus carpio*, *Danio rerio*), amphibians (*Xenopus laevis*), reptiles (*Anolis carolinensis*), and mammals (*Homo sapiens*), employing Thioflavin T (ThT) fluorescence assays and Circular Dichroism (CD) spectroscopy. The overarching objective was to explore potential evolutionary patterns underlying interspecific differences in aggregation behaviour. By focusing on these often-overlooked isoforms in non-mammalian species, this work seeks to provide new insights into the structural diversity of synucleins and their evolutionary conservation across vertebrate lineages.

The results indicate that non-mammalian β-synucleins are less prone to aggregation compared with human β-synuclein, whereas γ-synucleins from all species displayed a greater propensity to aggregate. Furthermore, copper may modulate this process, potentially influencing both the rate and extent of aggregation.

## 2. Materials and Methods

### 2.1. Cloning and Production of Recombinant Proteins

Coding sequences for human β-syn (NM_001001502.3) and γ-syn (NM_003087.3) were obtained from OriGene Technologies, Inc. (Rockville, MD, USA) (plasmids #RC215165 and #RC204173). Coding sequences for β-syn (NM_001091980) and γ-syn (NM_001087359) from *X. laevis* were available from previous studies [71,73]. Coding sequences for β- and γ-syn from *C. carpio*, *D. rerio* and *A. carolinensis* were cloned by RT-PCR using total RNA extracted from biological specimens obtained in previous studies [70,80]. Primer design was based on publicly available sequences of β- and γ-syns deposited in GenBank: *C. carpio* (XM_019115422.2 and XM_042735530.1), *D. rerio* (NM_200969.1 and NM_001020652.1), *A. carolinensis* (XM_062970200.1 and XM_008114476.3). All β- and γ-syn isoforms were cloned into pGEX-2T vector using BamHI-EcoRI or BamHI-SmaI restriction sites to produce GST-fusion proteins. All plasmids were sequence-verified before transformation into *Escherichia coli* BL21(DE3) cells, which were grown in LB medium supplemented with ampicillin to an OD_600_ of 0.5–0.6, at which point GST-syn expression was induced with 0.1 mM IPTG at 37 °C for 2–4 h. Cells were harvested and stored at −80 °C until use. Cells were resuspended in lysis buffer (20 mM potassium phosphate, pH 7.0, containing 150 mM NaCl, 1 mM PMSF, 1 mg/mL lysozyme) and sonicated to obtain a lysate, which was clarified by centrifugation at 20,000× *g* for 20 min. All GST-syn isoforms were purified on GSH-Sepharose Fast Flow (GE Healthcare, Chicago, IL, USA) according to the manufacturer’s instructions. To remove the GST tag, the purified fusion protein was treated with thrombin (Cytiva, Chicago, IL, USA) for 2 h and repurified on GSH-Sepharose. Synuclein was recovered in the unbound and wash fractions. The purified protein was concentrated by ultrafiltration with Vivaspin (10 kDa) filters (Sartorius AG, Göttingen, Germany) and dialysed in 10 mM potassium phosphate buffer, pH 7.0, containing 50 mM Na_2_SO_4_. Protein content was measured spectrophotometrically. The purity and integrity of protein samples were assessed by SDS-PAGE on 15% polyacrylamide gels.

### 2.2. Aggregation Kinetics

Aggregation of synuclein (5 µM) was performed at 37 °C with shaking at 160 rpm, in the absence or presence of CuSO_4_ (100 µM). Samples were withdrawn at the indicated times for recording of CD spectra and fluorescence spectra after addition of ThT (10 µM). Fluorescence spectra were recorded on a Fluoromax Jobin Yvon spectrofluorimeter at 20 °C with a 0.4 cm × 1 cm cuvette (excitation along the 0.4 cm pathlength). Excitation was at 450 nm, and emission spectra were collected between 460 and 660 nm; the excitation and emission slit widths were 5 nm. CD spectra were recorded on a Jasco J-810 spectropolarimeter in the range 190–260 nm, with 0.1 cm cuvettes. All spectra are the average of at least four scans with buffer spectra subtracted. To improve reliability, the mean fluorescence intensity values at 478–492 nm and CD ellipticity values at 194.6–195.4 nm were used to construct the time course plots. Two independent experiments were performed with different time points to broaden the temporal range of analysis. Time points tested in both experiments are shown as mean ± SEM; time points tested in only one experiment are shown as individual normalised values. Fluorescence and CD data were analysed using GraphPad Prism, version 10.5.0 (GraphPad Software, San Diego, CA, USA), OriginPro 2018 (64-bit) SR1, version 9.5.1.195 (OriginLab Corporation, Northampton, MA, USA), or Microsoft Excel for Microsoft 365 MSO, Version 2507 (Microsoft Corporation, Redmond, WA, USA).

### 2.3. Amino Acid Sequence Analysis

Comparative sequence analysis of β- and γ-syn was carried out using Clustal Omega (https://www.ebi.ac.uk/jdispatcher/msa/clustalo, accessed on 30 May 2025). Secondary structure predictions were generated using the PSIPRED Protein Sequence Analysis Workbench (http://bioinf.cs.ucl.ac.uk/psipred/, accessed on 30 May 2025) [81].

## 3. Results and Discussion

### 3.1. Sequence Variation and Predicted Structural Features of Vertebrate β- and γ-Synucleins

Recombinant β- and γ-syns were expressed in *E. coli* BL21 (DE3) cells as GST-fusion proteins using pGEX-2T vectors (Figure S1). Following affinity purification and cleavage of the GST tag, the proteins were processed under native conditions, as described in the Materials and Methods, to ensure consistent in vitro conditions for evaluating aggregation kinetics and structural transitions.

**Figure 1:**
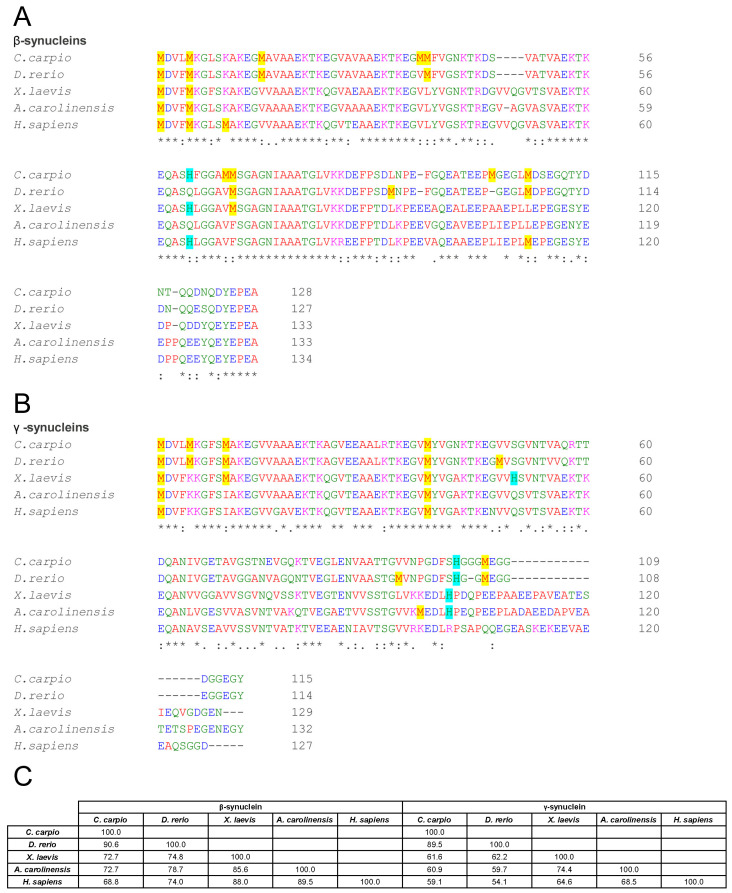
Comparative sequence analysis of β- and γ-synucleins across vertebrate species. (**A**,**B**) Multiple sequence alignment of β- (**A**) and γ- (**B**) syns amino acid sequences from *C. carpio*, *D. rerio*, *X. laevis*, *A. carolinensis*, and *H. sapiens*. Asterisks denote identical residues, double dots indicate conserved substitutions (similar polarity or size), and dots denote semi-conserved substitutions. Methionines are highlighted in yellow and histidines in light blue. (**C**) Matrix showing the percentage identity among β-syns and among γ-syns. Alignments and identity scores were generated using Clustal Omega.

Although synucleins are generally conserved across vertebrates, multiple sequence alignments revealed marked interspecies differences. For β-syn, sequence identity relative to the human isoform was 68.8% for *C. carpio*, 74.0% for *D. rerio*, 88.0% for *X. laevis*, and 89.5% for *A. carolinensis* (Figure 1A,C). A particularly interesting variation was observed at position 66 in carp β-syn, where methionine aligns with valine 70 in the human isoform. Notably, the V70M substitution in human β-syn has been associated with DLB [82] and shown to accelerate fibril formation relative to the wild-type protein [83]. This finding suggests that carp β-syn may exhibit altered aggregation dynamics compared with other non-mammalian orthologues.

γ-Syn exhibited greater divergence, with identity values of 59.1% (*C. carpio*), 54.1% (*D. rerio*), 64.6% (*X. laevis*), and 68.5% (*A. carolinensis*) relative to human γ-syn (Figure 1B,C). These differences may influence the secondary structure, aggregation propensity, and functional properties of the proteins.

**Figure 2:**
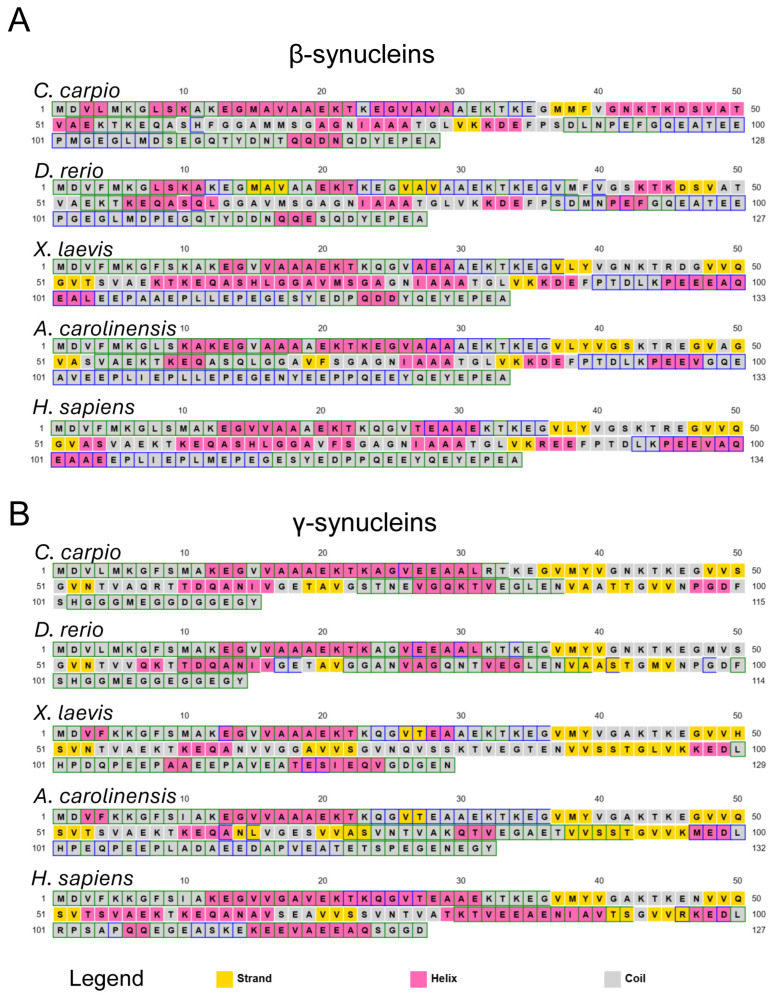
Predicted secondary structure of vertebrate synucleins. Secondary structure prediction for β- (**A**) and γ- (**B**) syns from *C. carpio*, *D. rerio*, *X. laevis*, *A. carolinensis*, and *H. sapiens*, obtained using the PSIPRED Protein Sequence Analysis Workbench (http://bioinf.cs.ucl.ac.uk/psipred/ (accessed on 30 May 2025) [81]. Predicted coils are shown in grey, α-helices in pink, and β-strands in yellow. More detailed cartoon versions are shown in Figures S2 and S3.

To investigate potential structural implications of these sequence variations, secondary structure predictions were conducted using the PSIPRED Protein Sequence Analysis Workbench [81] (Figure 2, Figures S2 and S3). The results revealed a largely conserved structural architecture across species. Predicted β-syns displayed a predominance of random-coil regions, interspersed with α-helices and a few short β-strand elements (Figure 2A and Figure S2).

At the N-terminal region (residues 1–36), no β-strands were predicted in any species except *D. rerio*, which showed a short β-sheet spanning residues 15–28. In the C-terminal region, no β-strands were predicted beyond residue 86 in most species; in *D. rerio*, β-sheet content was absent beyond residue 49 (Figure 2A and Figure S2). These computational patterns suggest that sequence divergence may translate into subtle differences in local folding propensity and potentially impact aggregation behaviour.

Similar observations were made for γ-syns (Figure 2B and Figure S3), which displayed dominant random coil conformations with intermittent α-helices and fewer β-strands, mostly confined to the central region (residues 36–96). Notably, *X. laevis* and *A. carolinensis* γ-syns contained short β-sheet segments at residues 26–27. The lack of β-strand elements at both N- and C-termini was consistent across species. These findings indicate species-specific secondary structure propensities that could be linked to distinct aggregation mechanisms.

Overall, the sequence divergence and predicted structural variability point to inherent differences in the folding landscapes of vertebrate β- and γ-syns. Such differences are likely to modulate their intrinsic tendency to undergo β-sheet conversion and subsequent fibril formation.

To validate this hypothesis, all recombinant synucleins were subjected to in vitro aggregation assays under controlled conditions. Proteins were incubated at 37 °C with constant agitation, in the presence or absence of CuSO_4_ (100 µM), a known aggregation modulator [84]. Aliquots were collected over time and analysed via ThT fluorescence and CD spectroscopy (Figure 3, Figure 4, Figure 5 and Figure 6 and Figures S4–S17). ThT fluorescence intensity at 480 nm was used as a sensitive marker for amyloid β-sheet content, while far-UV CD spectra confirmed conformational states.

For interpretative clarity, the aggregation timeline was subdivided into three temporal windows: phase I (0–50 h), phase II (51–100 h), and phase III (101–160 h), which correspond to lag, growth and plateau phases. These intervals facilitated comparisons of aggregation kinetics and structural transitions among species and treatment conditions.

### 3.2. β-Synucleins: Human β-Syn Aggregates Under In Vitro Conditions, Whereas Non-Mammalian Orthologues Remain Stable

**Figure 3:**
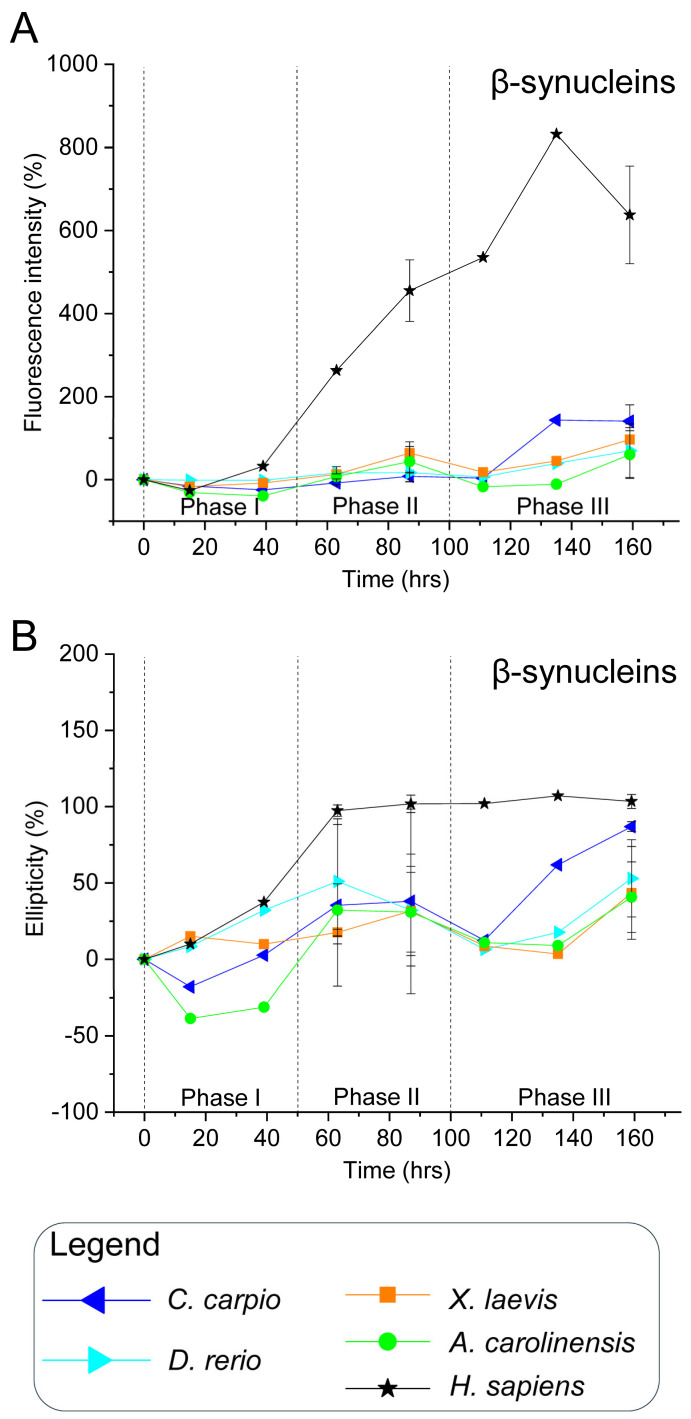
In vitro aggregation kinetics of β-synucleins from various vertebrate species. (**A**) Time-resolved Thioflavin T (ThT) fluorescence profiles indicating β-sheet formation. Fluorescence intensity at 480 nm (*y*-axis) is expressed as a percentage relative to the fluorescence measured at 0 h for each protein, and is plotted against incubation time (*x*-axis, hours). (**B**) Circular dichroism (CD) ellipticity at 195 nm (*y*-axis), expressed as a percentage relative to the initial value at 0 h for each protein, reflecting secondary structure transitions over time (*x*-axis). Time points analysed: 0, 15, 39, 63, 87, 111, 135, and 159 h. Data are from two independent experiments performed with different time points to broaden the temporal range of analysis (Figures S8 and S9). Time points 63, 87, and 159 h, tested in both experiments, are shown as mean ± SEM; all other time points, tested in only one experiment, are shown as individual normalised values. Symbols denote the species analysed: *C. carpio* (blue left-pointing triangles), *D. rerio* (light blue right-pointing triangles), *X. laevis* (orange squares), *A. carolinensis* (green circles), and *H. sapiens* (black stars).

ThT fluorescence analysis of human β-syn revealed a progressive increase in intensity from the end of phase I, sustained throughout the remainder of the incubation period (Figure 3A and Figure S8). This kinetic profile suggests a gradual conformational transition towards β-sheet-rich structures, indicative of amyloid-like aggregate formation. These observations were corroborated by far-UV CD spectroscopy, which showed a time-dependent increase in ellipticity at 195 nm (Figure 3B and Figure S9). Although no notable changes were observed during phase I, both fluorescence and ellipticity consistently increased from phase II onwards, reflecting a shift in secondary structure towards β-sheet content.

These findings demonstrate that human β-syn, despite lacking the non-amyloid-β component (NAC) domain found in α-syn, can aggregate under the present in vitro conditions, in agreement with previous reports [84].

In contrast, non-mammalian β-syn orthologues (*C. carpio*, *D. rerio*, *X. laevis*, and *A. carolinensis*) maintained low ThT fluorescence throughout incubation, with only marginal increases at the final stages (Figure 3A and Figure S8). CD measurements similarly showed limited conformational changes: ellipticity at 195 nm rose modestly during phase II and more markedly at the end of phase III (Figure 3B and Figure S9).

These data indicate a significantly lower aggregation propensity in non-mammalian β-syn compared with the human isoform. The delayed or minimal transitions may reflect either slower aggregation kinetics or an intrinsically reduced capacity to adopt β-sheet conformations. Extended incubation studies may distinguish between these possibilities.

Among the non-mammalian β-syns, *C. carpio* exhibited a comparatively higher increase in CD signal during phase III, suggesting greater conformational rearrangement (Figure 3B). This observation may be linked to the presence of methionine at position 66, potentially promoting structural transitions that facilitate aggregation.

These interspecies differences likely arise from sequence divergence, which may critically modulate protein folding and aggregation behaviour. It is plausible that the lower aggregation propensity of non-mammalian β-syns reflects evolutionary adaptations conferring functional or structural resilience. Indeed, synuclein function is closely associated with protein conformation: in human α-syn, α-helical structures mediate membrane binding and vesicle trafficking [85], while β-sheet-rich conformations underpin the formation of insoluble and potentially neurotoxic aggregates [36,86].

Thus, the differing abilities of β-syn orthologues to adopt β-sheet structures may reflect evolutionary divergence in synuclein function across vertebrate lineages. Furthermore, the synuclein gene repertoire varies significantly among species; for example, the genome of *D. rerio* lacks an α-syn gene [87,88], suggesting that β- and γ-syn isoforms may fulfil compensatory or species-specific roles [89].

### 3.3. γ-Synucleins: All Orthologues Undergo β-Sheet Transitions with Species-Specific Aggregation Dynamics

**Figure 4:**
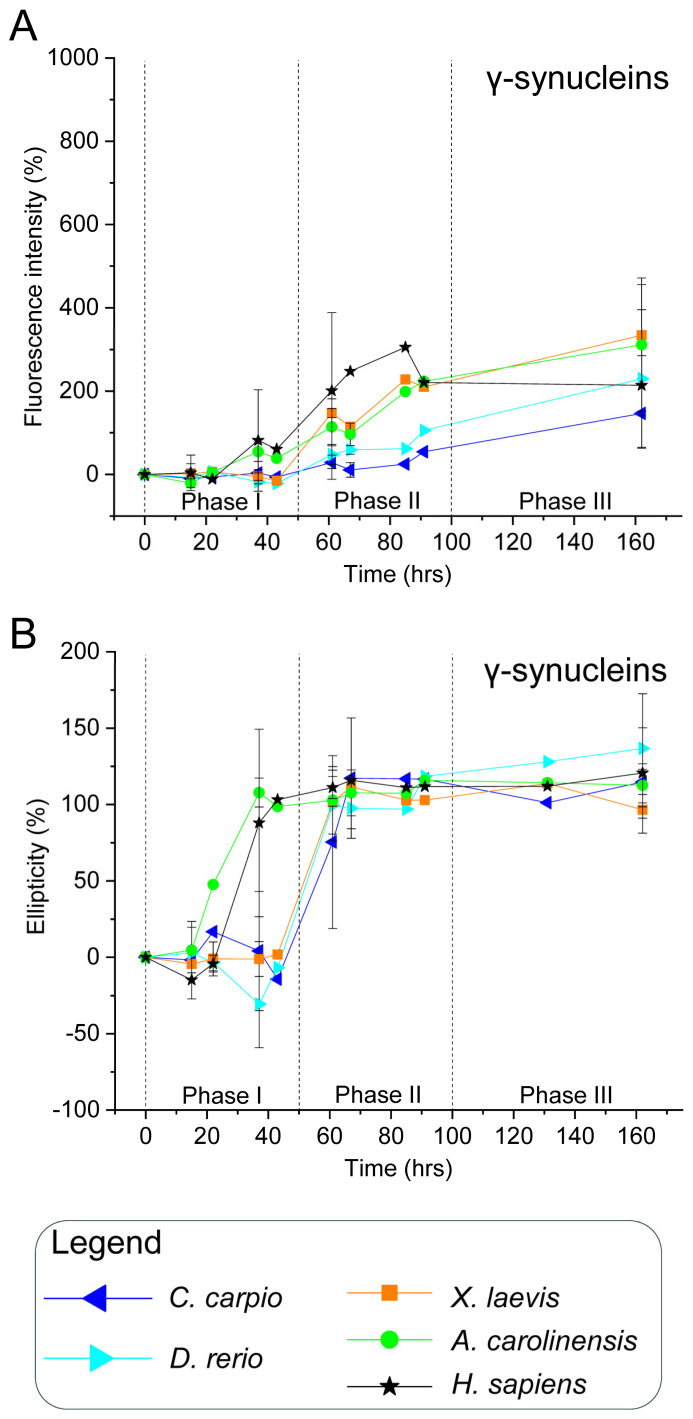
In vitro aggregation kinetics of γ-synucleins. (**A**) ThT fluorescence profiles and (**B**) CD ellipticity showing time-dependent aggregation and conformational changes in γ-syns. As in Figure 3, ThT fluorescence at 480 nm and CD signal at 195 nm are expressed as percentages relative to values at time 0 for each protein, and plotted over time. Measurements were taken at the following time points: 0, 15, 22, 37, 43, 61, 67, 85, 91, 131, and 162 h. Data are from two independent experiments performed with different time points to broaden the temporal range of analysis (Figures S10 and S11). Time points 15, 37, 61, 67 and 162 h, tested in both experiments, are shown as mean ± SEM; all other time points, tested in only one experiment, are shown as individual normalised values. Species and corresponding symbols are the same as those described in Figure 3.

ThT fluorescence analysis of human γ-syn revealed an early increase during phase I, which intensified through phases II and III (Figure 4A and Figure S10), indicating a strong propensity for β-sheet formation and aggregate development. CD spectra corroborated this trend, with rising ellipticity at 195 nm observed from ~37 h onwards, consistent with a rapid conformational transition to β-sheet structures (Figure 4B and Figure S11). These findings align with earlier reports that γ-syn can adopt β-sheet conformations and, under certain conditions, form fibrillar aggregates [90].

Non-mammalian γ-syn displayed similar behaviour, with increases in both ThT fluorescence and CD ellipticity beginning already in phase I or phase II (Figure 4, Figures S10 and S11). While all orthologues underwent β-sheet transitions, the kinetics and amplitude of these changes varied across species. Notably, *A. carolinensis* and *X. laevis* γ-syns showed more rapid and pronounced alterations than teleosts, suggesting species-specific modulation of aggregation dynamics.

### 3.4. Copper (CuSO_4_) Reduces Human β-Syn Aggregation and Modulates γ-Syn Dynamics in a Species-Specific Manner

Copper, an essential trace element, functions as a cofactor for numerous enzymes [91] and modulates folding and aggregation of prion and prion-like proteins, including synucleins [36].

Although most studies have focused on copper interactions with α-syn, available data indicate that β- and γ-syns also bind Cu^2+^ due to sequence similarity: all three human isoforms bind Cu^2+^ in a 1:1 stoichiometry, with γ-syn showing the highest affinity (picomolar), followed by α- and β-syn (nanomolar) [92].

In human β-syn, two primary Cu^2+^ binding sites are known: the N-terminal Met1/Met5 site and a His65-centred site [92]. Met10 is not directly involved in Cu^2+^ coordination but facilitates Cu^2+^ reduction, explaining the higher Cu^+^ affinity observed for β-syn compared with α-syn [54,93,94].

Sequence alignments (Figure 1A) show that Met1 and Met5 are conserved across all examined species, while Met10 is absent in non-mammalian orthologues. His65 is conserved in *C. carpio* (as His61) and *X. laevis*, but is absent in *D. rerio* and *A. carolinensis*, suggesting potential differences in copper-binding properties and aggregation susceptibility across taxa.

The effects of metal binding on prion and prion-like proteins such as PrP^C^ and α-syn have been studied using a wide range of metal concentrations. While some works employed high levels (0.5–5.0 mM) [50,51,80,95], others used more physiologically relevant conditions [96,97]. Following the latter approach, we investigated the effect of 100 µM CuSO_4_ on vertebrate β- and γ-syns.

**Figure 5:**
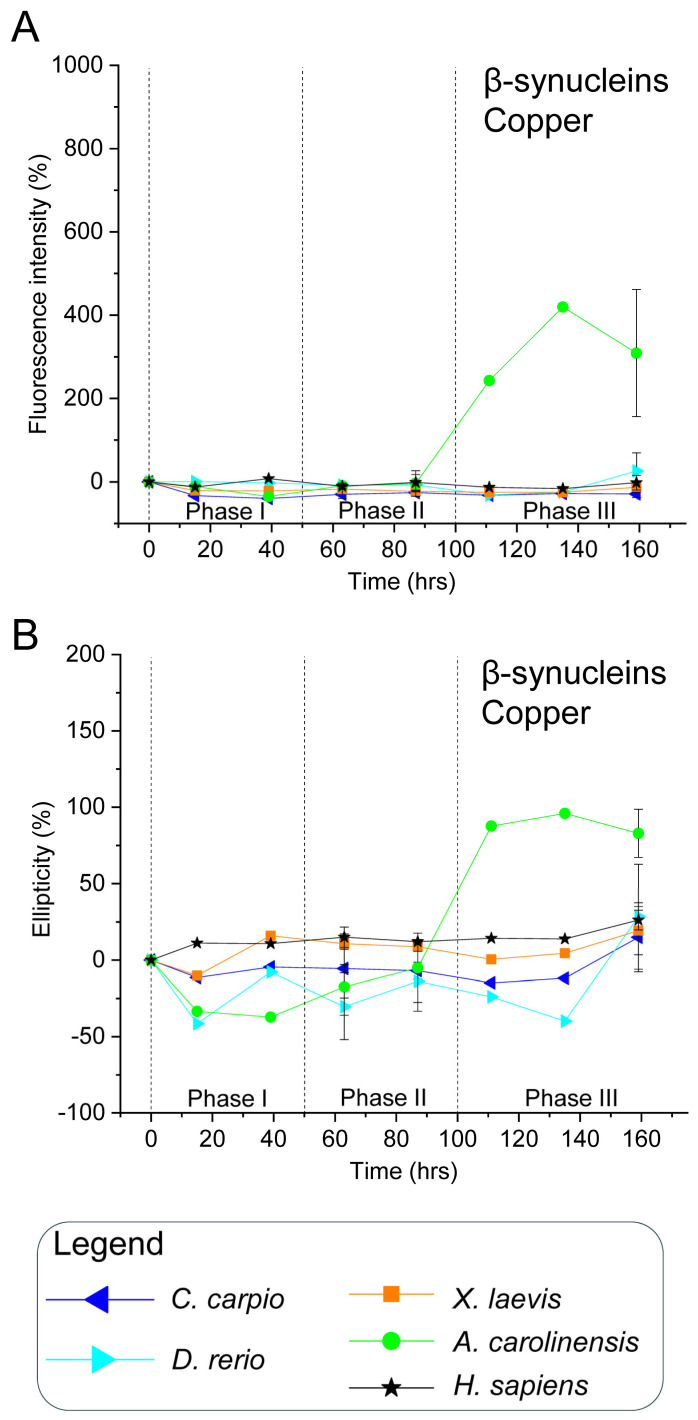
In vitro aggregation kinetics of β-synucleins in the presence of copper. **(A**) ThT fluorescence profiles and (**B**) CD ellipticity recorded in the presence of 100 μM CuSO_4_. ThT fluorescence intensity at 480 nm and CD signal at 195 nm are expressed as percentages relative to values at 0 h for each protein, and plotted over time. Time points, species, and symbol coding are the same as in Figure 3. Data shown are representative of two independent experiments (Figures S12 and S13).

For human β-syn, Cu^2+^ caused negligible increases in ThT fluorescence and CD signal across incubation (Figure 5, Figures S12, S13 and S16), indicating that copper inhibits or delays β-sheet formation and aggregation at the tested concentrations, likely due to occupancy of metal-binding sites [54,93]. These results differ from earlier reports where copper promoted α- and β-syn aggregation [98,99], possibly due to the much higher protein (70 µM) and Cu^2+^ (5 mM) concentrations used in those studies compared with the present work (5 µM β-syn, 100 µM Cu^2+^). Future studies are needed to clarify how different copper concentrations may modulate the aggregation propensity of vertebrate synuclein isoforms.

In non-mammalian β-syns—which displayed minimal aggregation in the absence of copper—CuSO_4_ produced no significant changes in ThT or CD signals (Figure 5, Figures S12 and S13), suggesting copper does not promote β-sheet formation and may play a protective role akin to that in the human isoform. However, *A. carolinensis* β-syn exhibited a pronounced increase in ThT fluorescence and CD ellipticity during phase III upon copper exposure. This species-specific effect may stem from unique copper–protein interactions and vulnerability to misfolding.

**Figure 6:**
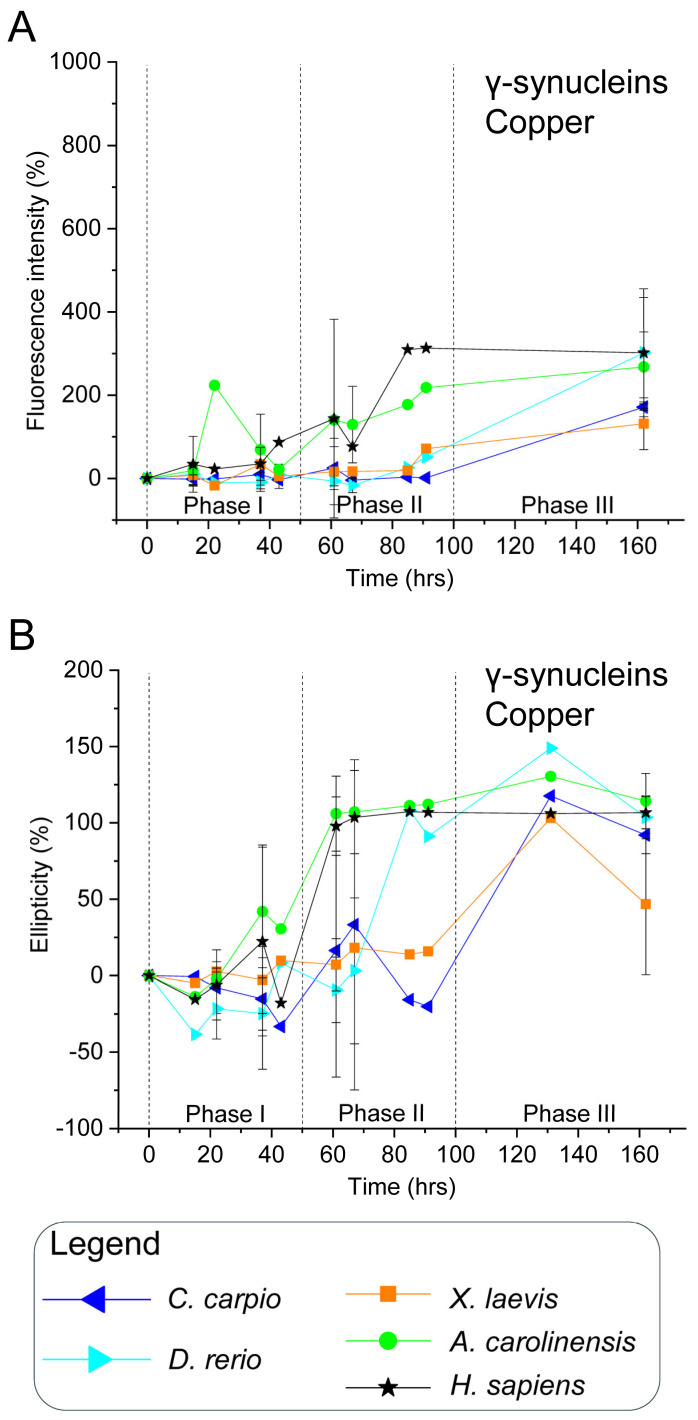
In vitro aggregation kinetics of γ-synucleins in the presence of copper. (**A**) ThT fluorescence profiles and (**B**) CD ellipticity recorded in the presence of 100 μM CuSO_4_. Axis labelling and data normalisation follow the same criteria described in Figure 3. Measurements were taken at the same time points reported in Figure 4. Species and corresponding symbols are as indicated in Figure 3. Data shown are representative of two independent experiments (Figures S14 and S15).

Although human γ-syn lacks Met5, Met10, and histidine residues, it retains Met1 (Figure 1B), and previous data suggest high-affinity copper binding [92]. In this study, ThT and CD analyses showed that human γ-syn maintained elevated fluorescence and ellipticity during phases II and III, even in the presence of Cu^2+^ (Figure 6, Figures S14, S15 and S17), indicating aggregation proceeds despite copper. However, a delayed ellipticity rise in early phases, coupled with a sharper ThT peak in phase II, suggests copper may transiently slow early conformational changes without altering the overall aggregation trajectory.

Sequence comparisons of non-mammalian γ-syn revealed species-specific variation in methionine and histidine residues in the N-terminal region (Figure 1B): *C. carpio* and *D. rerio* contain Met1, Met5, and Met10; *X. laevis* retains Met1 and Met10; while *A. carolinensis* and humans present only Met1. Histidine residues occur at distinct positions: *X. laevis* (His50, His101), *C. carpio* and *D. rerio* (His102), and *A. carolinensis* (His101), potentially affecting copper affinity and aggregation outcomes.

In non-mammalian γ-syn, copper elicited effects similar to those observed for the human protein. High ThT fluorescence and CD signals were recorded in phase III (Figure 6, Figures S14, S15 and S17), confirming that aggregation proceeds despite Cu^2+^. Interestingly, *X. laevis* γ-syn showed reduced fluorescence and ellipticity compared with the control, suggesting partial inhibition. In all species, copper appeared to delay the onset of ThT and CD signal increases, supporting the notion that interspecies differences in primary sequence modulate copper’s influence on γ-syn conformational transitions and aggregation kinetics.

## 4. Conclusions

This study provides novel comparative insights into the conformational transitions and aggregation behaviour of β- and γ-syns across diverse vertebrate taxa. Despite their evolutionary conservation, our results reveal marked species-specific differences in both aggregation propensity and copper sensitivity, suggesting divergent physiological roles and adaptive trajectories.

The robust aggregation of human β- and γ-syns under in vitro conditions—compared with the more limited structural transitions of non-mammalian isoforms—may reflect evolutionary pressures associated with increased nervous system complexity or longevity. Notably, the ability of human β-syn to form β-sheet-rich aggregates despite lacking the NAC domain highlights the role of subtle sequence determinants and environmental modulators such as metal ions in influencing structural plasticity.

Copper exposure elicited differential effects across taxa, exerting a protective, anti-aggregative influence on human β-syn, while enhancing aggregation in *A. carolinensis*. These observations point to taxon-specific mechanisms of metal-mediated modulation, potentially shaped by differences in copper-binding motifs and cellular stress responses. Further studies varying copper concentrations will be required to clarify whether dose-dependent effects influence the aggregation propensity of vertebrate synuclein isoforms.

Furthermore, the pronounced aggregation of γ-syn—even in species lacking α-syn, such as *D. rerio*—raises the possibility that the remaining synuclein isoforms may assume broader structural and functional roles in such organisms, compensating for the absence of α-syn through enhanced conformational flexibility or interaction with membrane components.

While the present study relied on ThT fluorescence and CD spectroscopy, additional methodologies such as transmission electron microscopy (TEM) or atomic force microscopy (AFM) will be important to confirm the amyloid nature and morphology of any fibrils formed. Likewise, direct copper-binding assays (e.g., ITC or MST) and site-directed mutagenesis approaches—such as testing clinically relevant substitutions exemplified by human V70M—will be required to substantiate and extend the present findings.

Altogether, our results underscore the importance of studying protein dynamics in an evolutionary context. The species-specific aggregation patterns and responses to metal ions observed here may provide important clues to the physiological diversity of synucleins and their involvement in neurodegenerative disorders. Future studies employing these complementary biophysical and genetic approaches, together with cellular and in vivo models, will be essential to determine the functional significance of the interspecies differences identified here and their relevance to synucleinopathies.

## Data Availability

The data presented in this study are available in this article and the Supplementary Materials. Further inquiries can be directed to the corresponding authors.

## Supplementary Materials

The following supporting information can be downloaded at https://www.mdpi.com/article/10.3390/biom15091231/s1, Figure S1: SDS-PAGE analysis of purified recombinant β- and γ-synucleins from representative vertebrate species. Recombinant proteins were separated on a 15% SDS-PAGE gel and visualised by Coomassie Brilliant Blue staining. β-syn from *A. carolinensis* (1), *C. carpio* (2), *D. rerio* (3), *H. sapiens* (4), *X. laevis* (5)*;* and γ-syn from *A. carolinensis* (6), *C. carpio* (7), *D. rerio* (8), *H. sapiens* (9) are shown. Lane 10 shows molecular weight markers (kDa); Figure S2: Predicted secondary structure of vertebrate β-synucleins. Secondary structure predictions of β-syn sequences from: (A) *C. carpio*, (B) *D. rerio*, (C) *X. laevis*, (D) *A. carolinensis*, and (E) *H. sapiens*, obtained via the PSIPRED Protein Structure Prediction tool (http://bioinf.cs.ucl.ac.uk/psipred/) [81]. Each panel displays: “AA” (amino acid sequence), “Pred” (predicted secondary structure: H = α-helix in pink, E = β-strand in yellow, C = coil in grey), “Cart” (cartoon summary), and “Conf” (prediction confidence, shown as blue bars); Figure S3: Predicted secondary structure of vertebrate γ-synucleins. Same layout and interpretation as Figure S2, applied to γ-syns from the same five species; Figure S4: ThT emission spectra monitoring β-sheet formation of β-synucleins. Time-resolved Thioflavin T fluorescence emission spectra (arbitrary units, A.U.) recorded for β-syns from five species—*C. carpio*, *D. rerio*, *X. laevis*, *A. carolinensis*, and *H. sapiens* (top to bottom rows). Samples were incubated at 37 °C in the absence (left panels) or presence (right panels) of 100 μM CuSO_4_. Emission was collected from 460 to 660 nm at the following time points: 0, 15, 39, 63, 87, 111, 135, and 159 h. Fluorescence enhancement near 480 nm reflects progressive β-sheet formation and aggregation; Figure S5: Far-UV CD spectra of β-synucleins during aggregation. CD spectra (190–260 nm) were recorded over time for the same species, conditions, and time points as in Figure S4. Ellipticity is expressed in millidegrees (mdeg); Figure S6: ThT emission spectra monitoring β-sheet formation of γ-synucleins. As in Figure S4, time-resolved Thioflavin T fluorescence emission spectra (A.U.) were recorded for γ-syns from five vertebrate species, during incubation at 37 °C in the absence (left panels) or presence (right panels) of 100 μM CuSO_4_. Emission was collected from 460 to 660 nm at the following time points: 0, 15, 22, 37, 38, 43, 61, 67, 85, 91, 131, and 162 h; Figure S7: Circular dichroism spectroscopy of γ-synucleins over time. Far-UV CD spectra collected across 190-260 nm were recorded for the same species, conditions and time points reported in Figure S6; Figure S8: In vitro aggregation kinetics of β-synucleins from various vertebrate species, monitored by time-resolved Thioflavin T (ThT) fluorescence. Fluorescence intensity at 480 nm (y-axis) is expressed as a percentage of the value measured at 0 h for each protein and plotted against incubation time (x-axis, hours), reflecting β-sheet formation. Data are from two independent experiments, each including different time points to extend the temporal range of analysis. For β-syn from *C. carpio* (A), *D. rerio* (B), *X. laevis* (C), *A. carolinensis* (D), and *H. sapiens* (E), measurements were taken at 0, 15, 39, 63, 87, and 159 h for experiment 1, and at 0, 63, 87, 111, 135, and 159 h for experiment 2. Upward- and downward-pointing triangles denote experiments 1 and 2, respectively. Panel F combines data from panels A–E: time points measured in both experiments are shown as mean ± SEM, whereas those from only one experiment are presented as individual normalized values. Symbols indicate species: *C. carpio* (blue left-pointing triangles), *D. rerio* (light blue right-pointing triangles), *X. laevis* (orange squares), *A. carolinensis* (green circles), and *H. sapiens* (black stars); Figure S9: In vitro aggregation kinetics of β-synucleins from various vertebrate species, monitored by circular dichroism (CD) spectroscopy. Ellipticity at 195 nm (y-axis) is expressed as a percentage of the value measured at 0 h for each protein and plotted against incubation time (x-axis, hours), reflecting secondary structure transitions. Data are from two independent experiments, each including different time points to extend the temporal range of analysis. Measurements were taken at 0, 15, 39, 63, 87, and 159 h for experiment 1, and at 0, 63, 87, 111, 135, and 159 h for experiment 2. Species, value definition, and symbol coding are as in Figure S8; Figure S10: In vitro aggregation kinetics of γ-synucleins from various vertebrate species, monitored by time-resolved ThT fluorescence. Fluorescence intensity at 480 nm (y-axis) is expressed as a percentage of the value measured at 0 h for each protein and plotted against incubation time (x-axis, hours), reflecting β-sheet formation. Data are from two independent experiments, each including different time points to extend the temporal range of analysis. Measurements were taken at 0, 15, 22, 37, 61, 67, and 162 h for experiment 1, and at 0, 15, 37, 43, 61, 67, 85, 91, and 162 h for experiment 2. Species, value definition, and symbol coding are as in Figure S8; Figure S11: In vitro aggregation kinetics of γ-synucleins from various vertebrate species, monitored by CD spectroscopy. Ellipticity at 195 nm (y-axis) is expressed as a percentage of the value measured at 0 h for each protein and plotted against incubation time (x-axis, hours), reflecting secondary structure transitions. Data are from two independent experiments, each including different time points to extend the temporal range of analysis. Measurements were taken at 0, 15, 22, 37, 61, 67, 131, and 162 h for experiment 1, and at 0, 15, 37, 43, 61, 67, 85, 91, and 162 h for experiment 2. Species, value definition, and symbol coding are as in Figure S8; Figure S12: In vitro aggregation kinetics of β-synucleins from various vertebrate species in the presence of copper, monitored by time-resolved ThT fluorescence. Fluorescence intensity at 480 nm (y-axis) is expressed as a percentage of the value measured at 0 h for each protein and plotted against incubation time (x-axis, hours), reflecting β-sheet formation. Measurements were recorded in the presence of 100 μM CuSO_4_. Data are from two independent experiments, each including different time points to extend the temporal range of analysis. Measurements were taken at 0, 15, 39, 63, 87, and 159 h for experiment 1, and at 0, 63, 87, 111, 135, and 159 h for experiment 2. Species, value definition, and symbol coding are as in Figure S8; Figure S13: In vitro aggregation kinetics of β-synucleins from various vertebrate species in the presence of copper, monitored by CD spectroscopy. Ellipticity at 195 nm (y-axis) is expressed as a percentage of the value measured at 0 h for each protein and plotted against incubation time (x-axis, hours), reflecting secondary structure transitions. Measurements were recorded in the presence of 100 μM CuSO_4_. Data are from two independent experiments, each including different time points to extend the temporal range of analysis. Measurements were taken at 0, 15, 39, 63, 87, and 159 h for experiment 1, and at 0, 63, 87, 111, 135, and 159 h for experiment 2. Species, value definition, and symbol coding are as in Figure S8; Figure S14: In vitro aggregation kinetics of γ-synucleins from various vertebrate species in the presence of copper, monitored by time-resolved ThT fluorescence. Fluorescence intensity at 480 nm (y-axis) is expressed as a percentage of the value measured at 0 h for each protein and plotted against incubation time (x-axis, hours), reflecting β-sheet formation. Measurements were recorded in the presence of 100 μM CuSO_4_. Data are from two independent experiments, each including different time points to extend the temporal range of analysis. Measurements were taken at 0, 15, 22, 37, 61, 67, and 162 h for experiment 1, and at 0, 15, 37, 43, 61, 67, 85, 91, and 162 h for experiment 2. Species, value definition, and symbol coding are as in Figure S8; Figure S15: In vitro aggregation kinetics of γ-synucleins from various vertebrate species in the presence of copper, monitored by CD spectroscopy. Ellipticity at 195 nm (y-axis) is expressed as a percentage of the value measured at 0 h for each protein and plotted against incubation time (x-axis, hours), reflecting secondary structure transitions. Measurements were recorded in the presence of 100 μM CuSO_4_. Data are from two independent experiments, each including different time points to extend the temporal range of analysis. Measurements were taken at 0, 15, 22, 37, 61, 67, 131, and 162 h for experiment 1, and at 0, 22, 37, 43, 61, 67, 85, 91, and 162 h for experiment 2. Species, value definition, and symbol coding are as in Figure S8; Figure S16: Comparative analysis of β-synuclein aggregation kinetics and conformational transitions in the absence and presence of copper. Plots show normalized Thioflavin T (ThT) fluorescence intensity at 480 nm (solid lines) and circular dichroism (CD) ellipticity at 195 nm (dashed lines) over time (x-axis, hours) for β-syns from: (A) *C. carpio*, (B) *D. rerio*, (C) *X. laevis*, (D) *A. carolinensis*, and (E) *H. sapiens*. Fluorescence and ellipticity values are expressed as percentages relative to each protein’s respective value at time 0 h. Open symbols indicate measurements in the absence of 100 μM CuSO_4_; filled symbols indicate the presence of copper. Data summarize those shown in Figure 3 and Figure 5, using the same time points: 0, 15, 39, 63, 87, 111, 135, and 159 h. Species and symbol coding as described in Figure 3; Figure S17: Comparative analysis of γ-synuclein aggregation kinetics and conformational transitions in the absence and presence of copper. As in Figure S8, ThT fluorescence (solid lines) and CD ellipticity (dashed lines) are shown over time for γ-syns from the same five species. Values are expressed as percentages relative to time 0 h for each protein. Data correspond to those presented in Figure 4 and Figure 6, with the same time points: 0, 15, 22, 37, 38, 43, 61, 67, 85, 91, 131, and 162 h. Open and filled symbols indicate conditions without and with 100 μM CuSO_4_, respectively. Species and symbol coding as in Figure 3.

## Abbreviations

The following abbreviations are used in this manuscript:

AD	Alzheimer’s disease
CD	Circular dichroism
AFM	Atomic force microscopy
DLB	Dementia with Lewy bodies
ITC	Isothermal titration calorimetry
MSA	Multiple system atrophy
MST	Microscale thermophoresis
NAC	Non-amyloid component
PD	Parkinson’s disease
TEM	Transmission electron microscopy
ThT	Thioflavin T
