# Supplementary figures and images for: Comparative Analysis of Aggregation of β- and γ-Synucleins in Vertebrates
Source: Biomolecules. 2025 Aug 26;15(9):1231. doi: 10.3390/biom15091231 (PMC12467708; doi:10.3390/biom15091231)

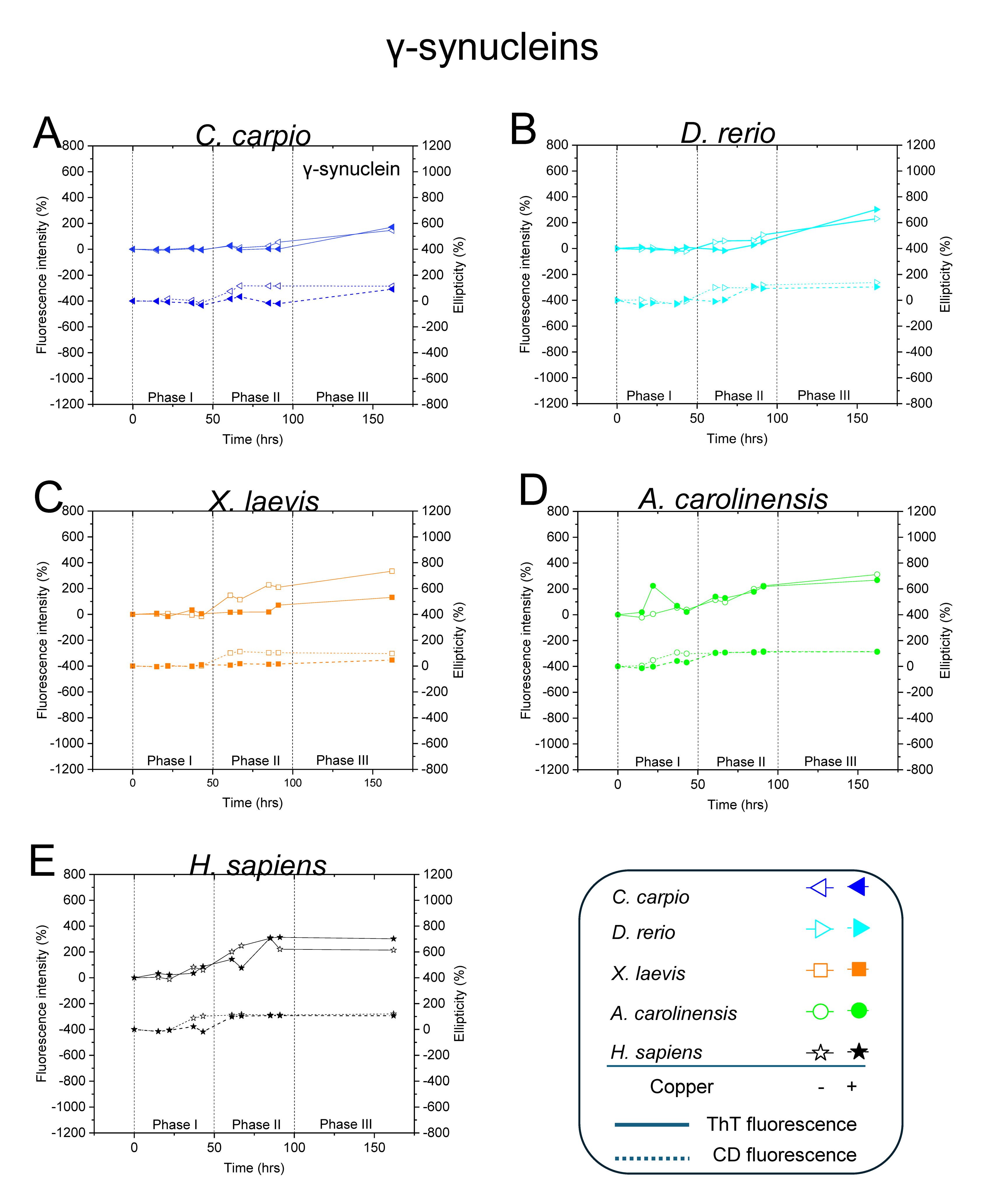

Supplement: Supplementary file 1 [file biomolecules-15-01231-s001.zip › Figure S17_600dpi (pixel-inch).tif]

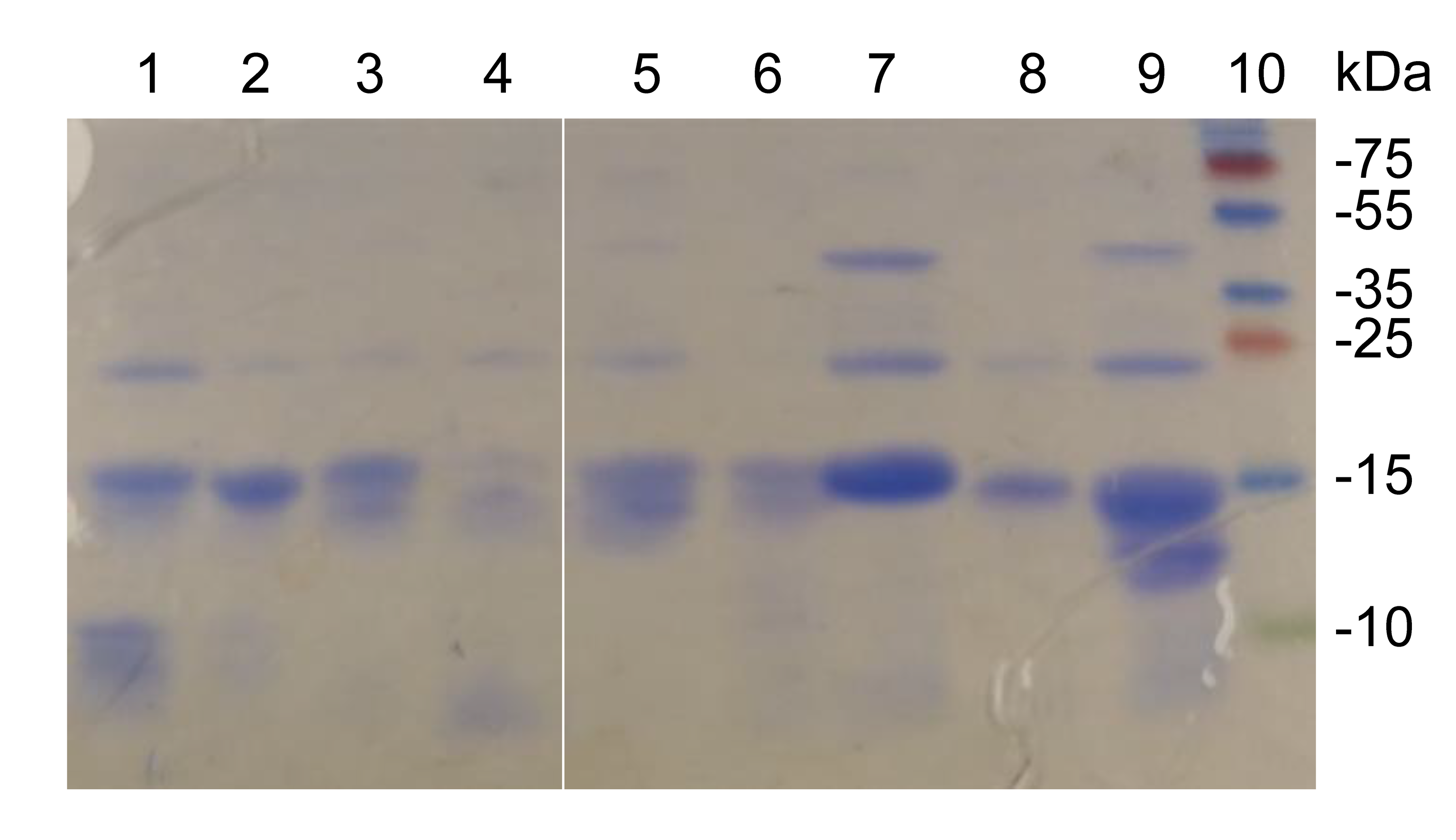

Supplement: Supplementary file 1 [file biomolecules-15-01231-s001.zip › Figure S1_600dpi (pixel-inch).tif]

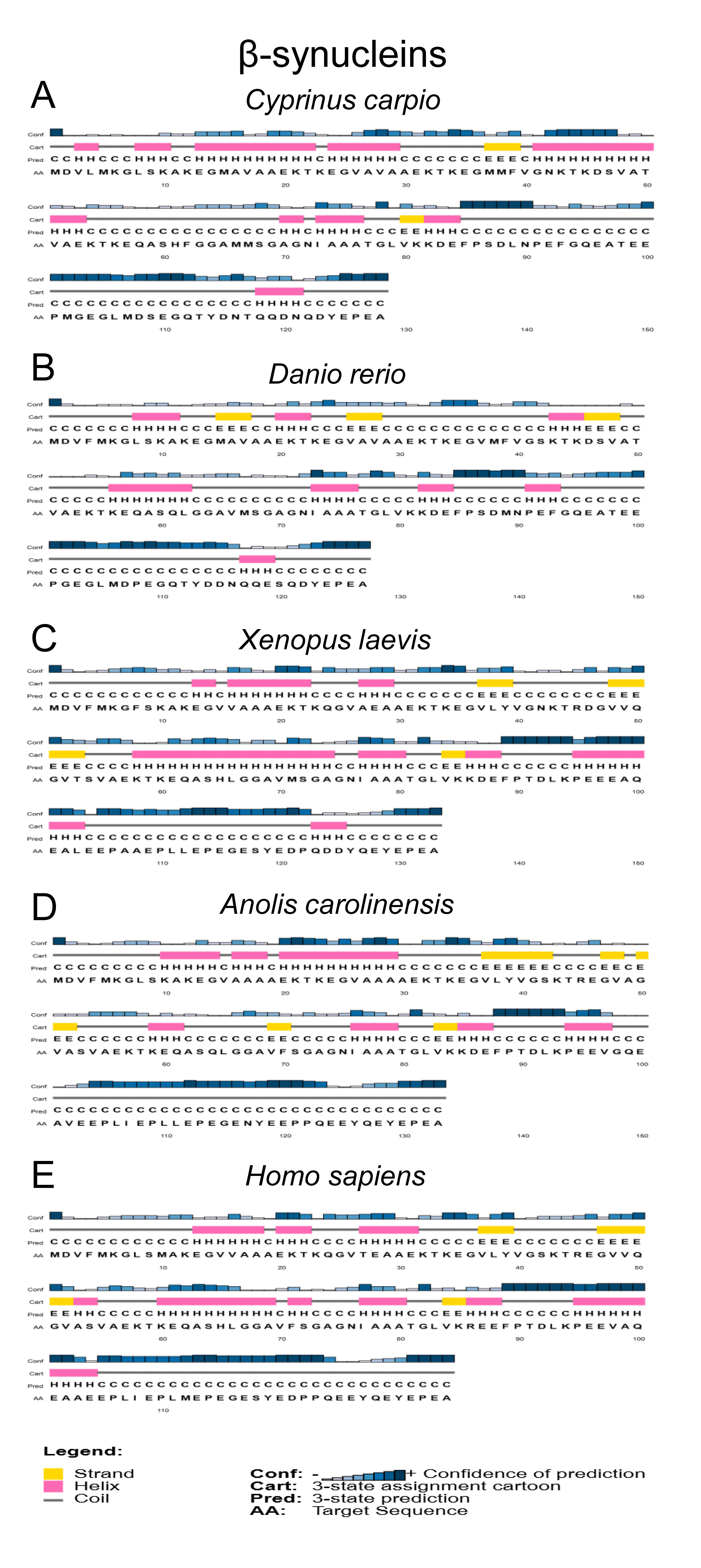

Supplement: Supplementary file 1 [file biomolecules-15-01231-s001.zip › Figure S2_600dpi (pixel-inch).tif]

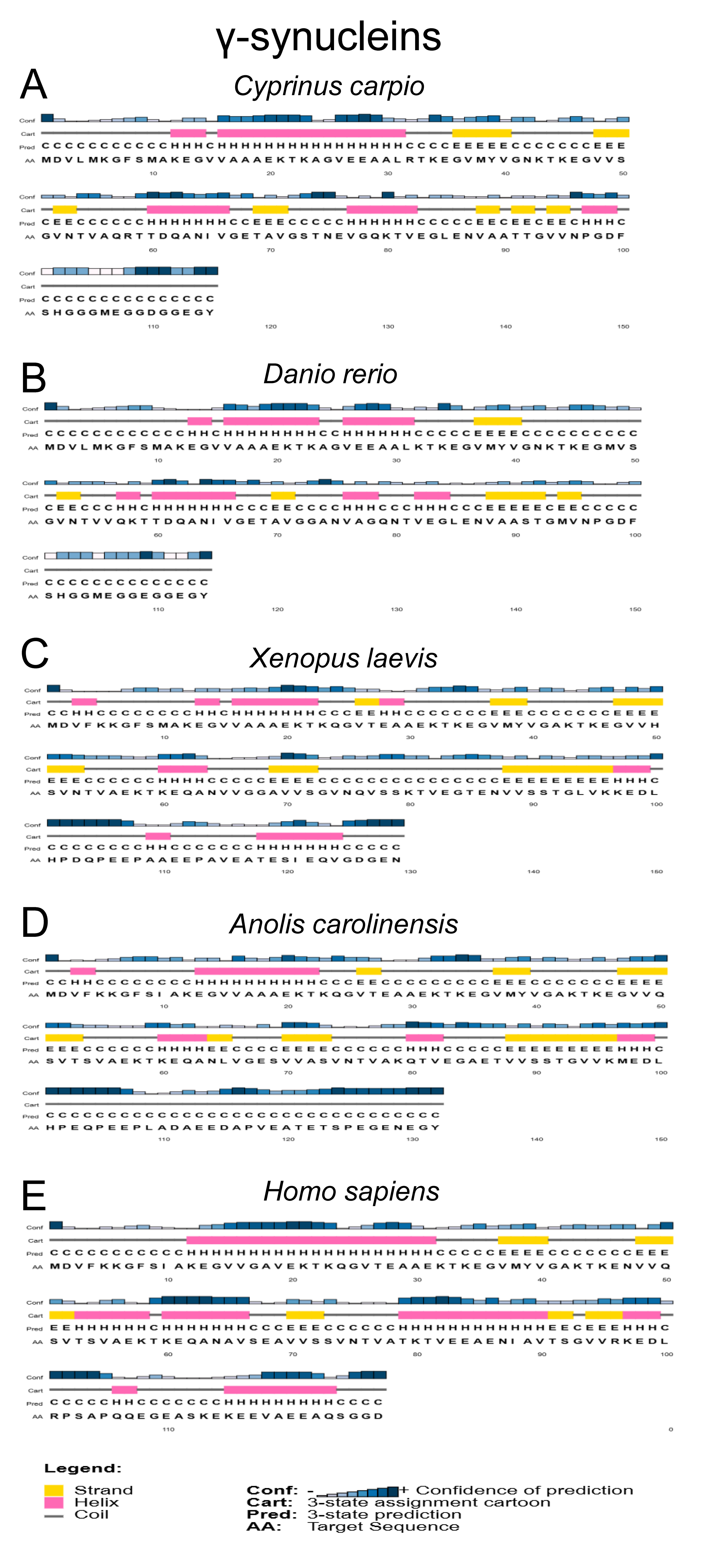

Supplement: Supplementary file 1 [file biomolecules-15-01231-s001.zip › Figure S3_600dpi (pixel-inch).tif]

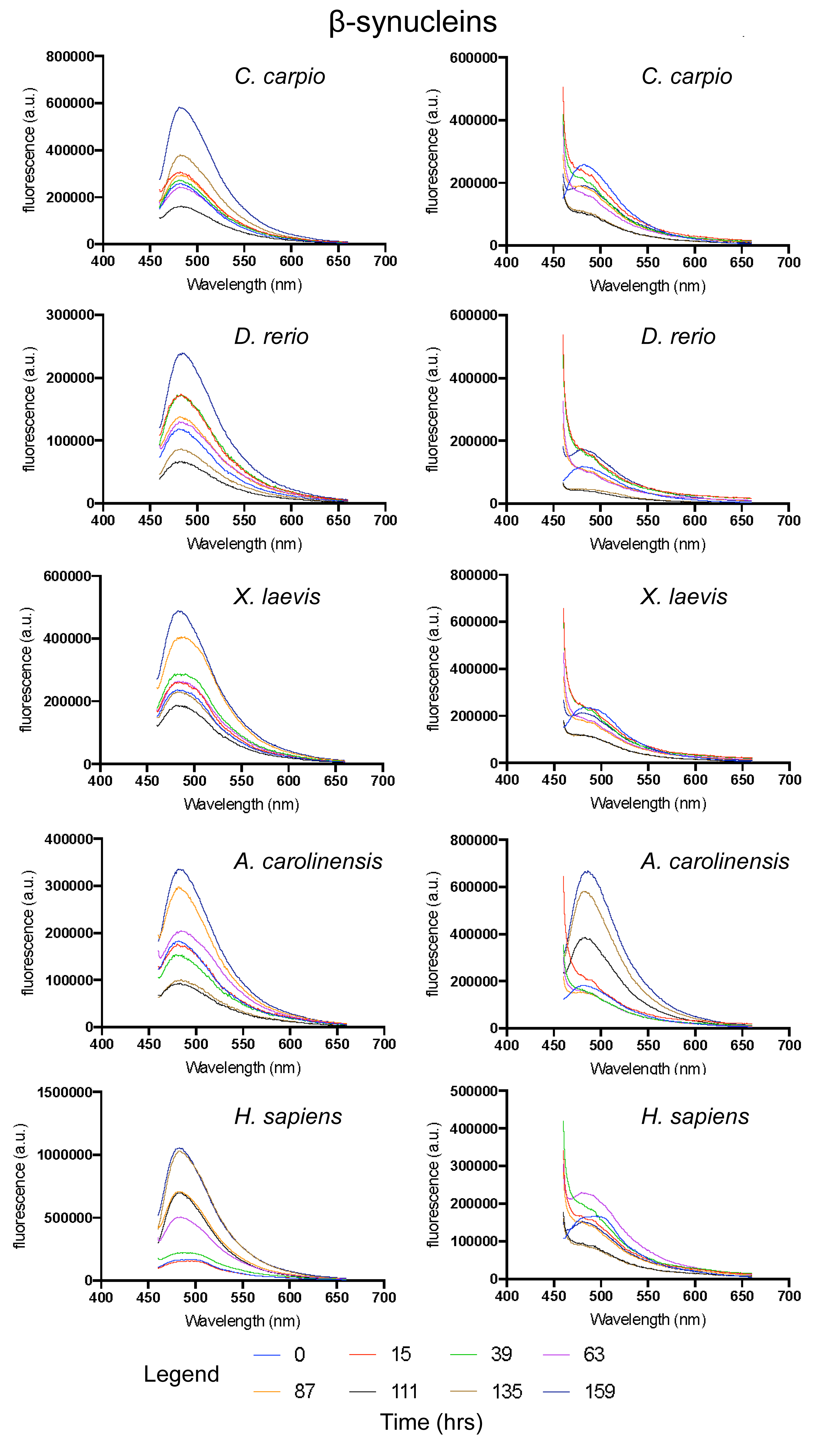

Supplement: Supplementary file 1 [file biomolecules-15-01231-s001.zip › Figure S4_600dpi (pixel-inch).tif]

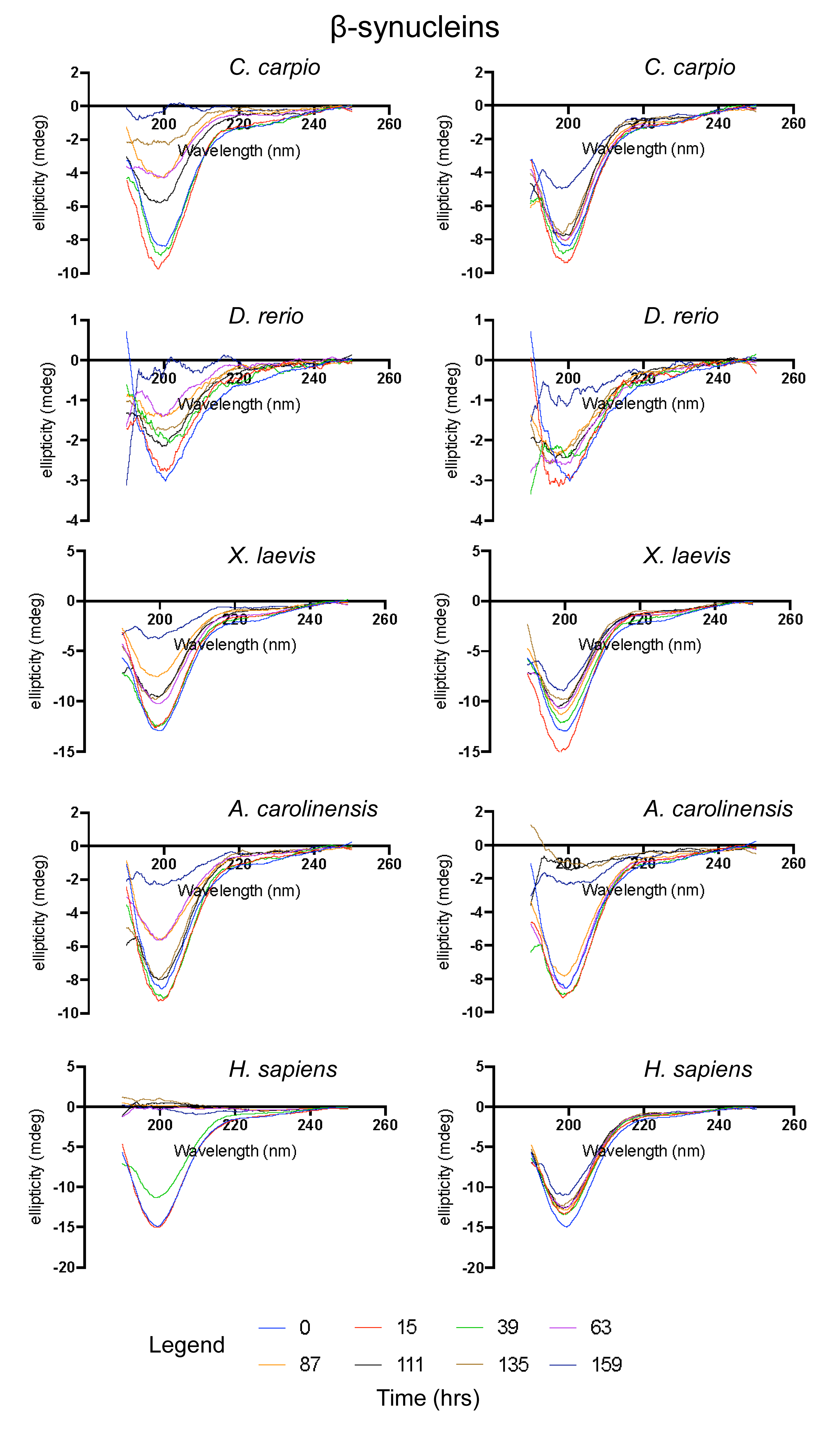

Supplement: Supplementary file 1 [file biomolecules-15-01231-s001.zip › Figure S5_600dpi (pixel-inch).tif]

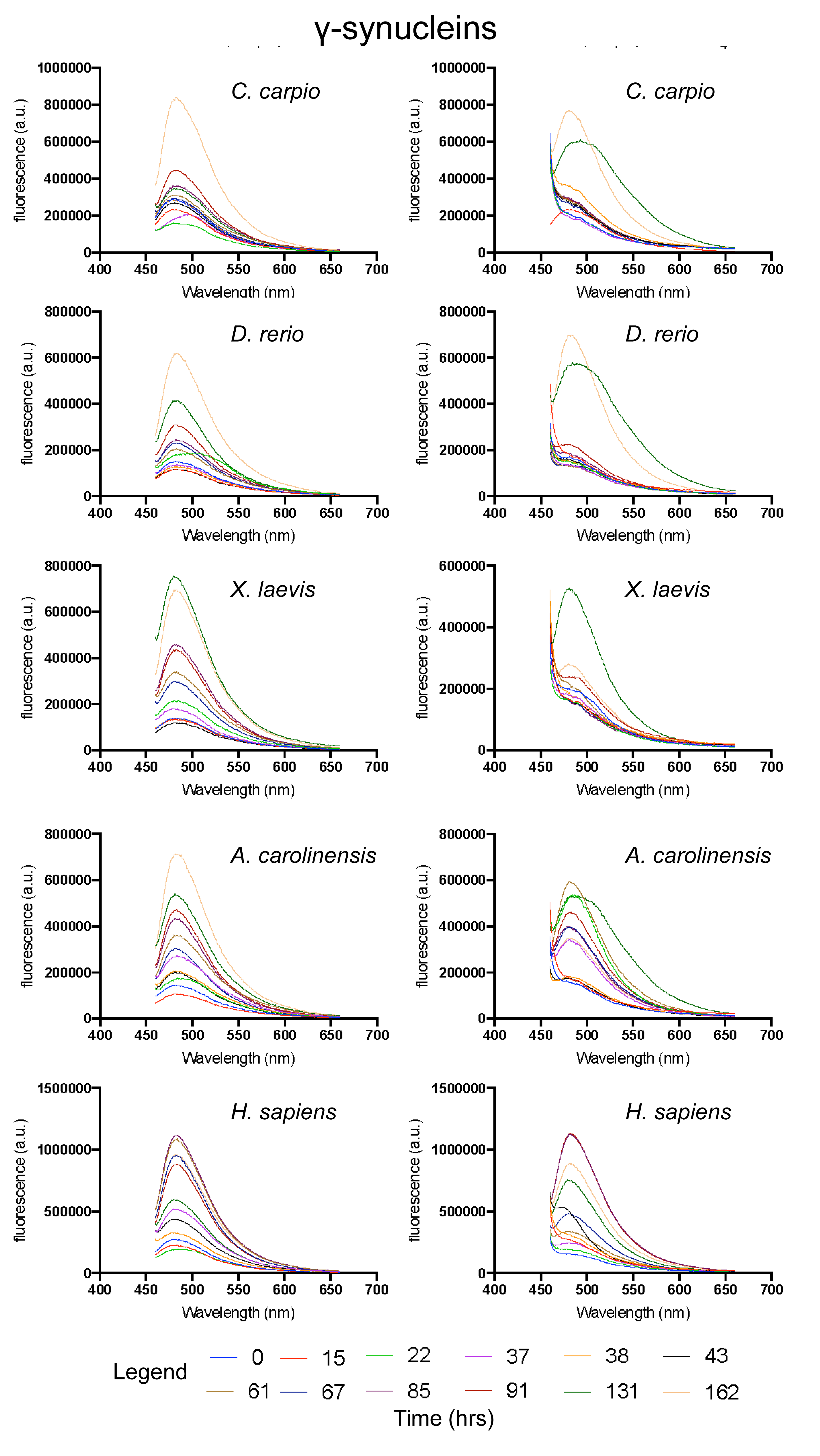

Supplement: Supplementary file 1 [file biomolecules-15-01231-s001.zip › Figure S6_600dpi (pixel-inch).tif]

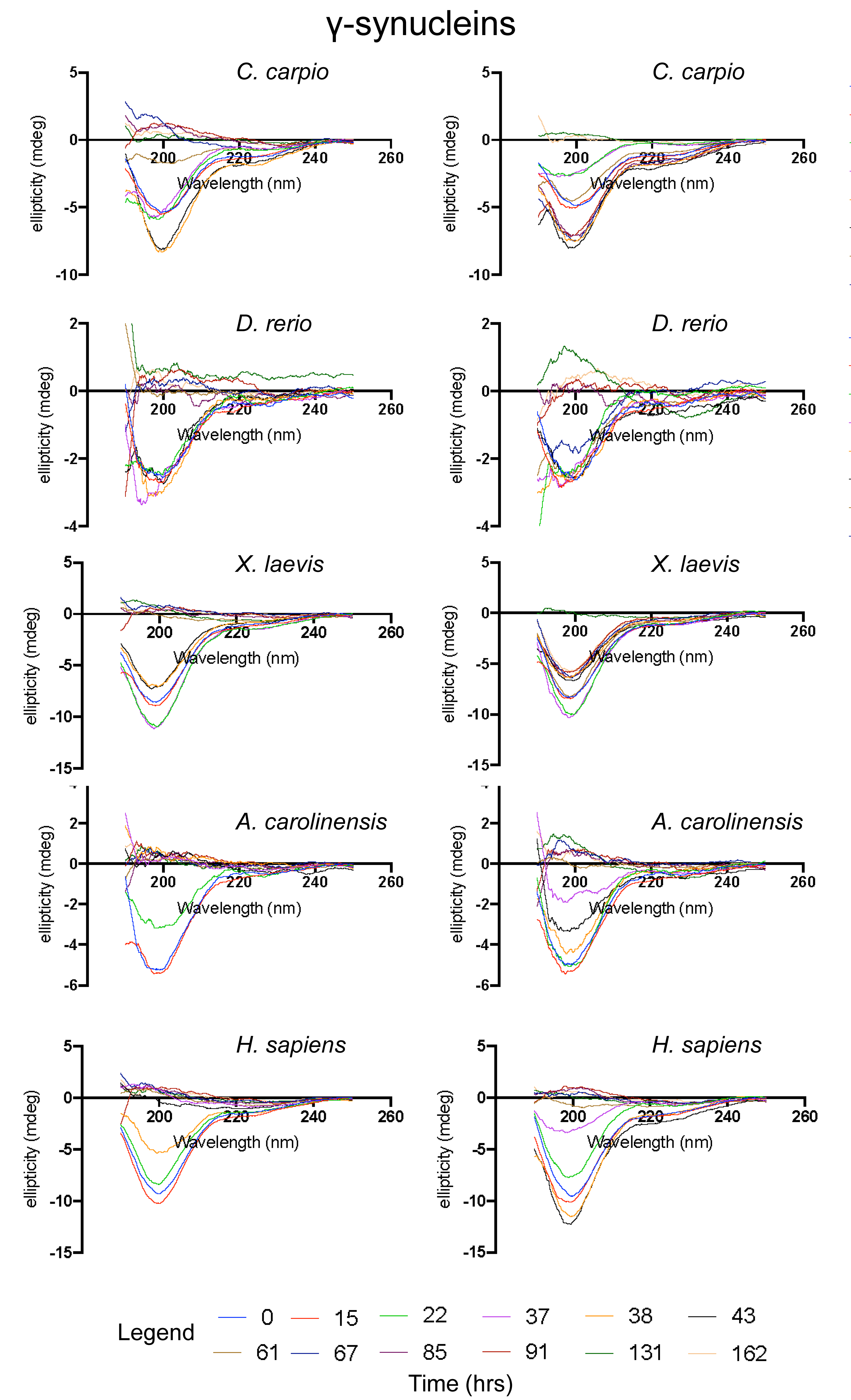

Supplement: Supplementary file 1 [file biomolecules-15-01231-s001.zip › Figure S7_600dpi (pixel-inch).tif]

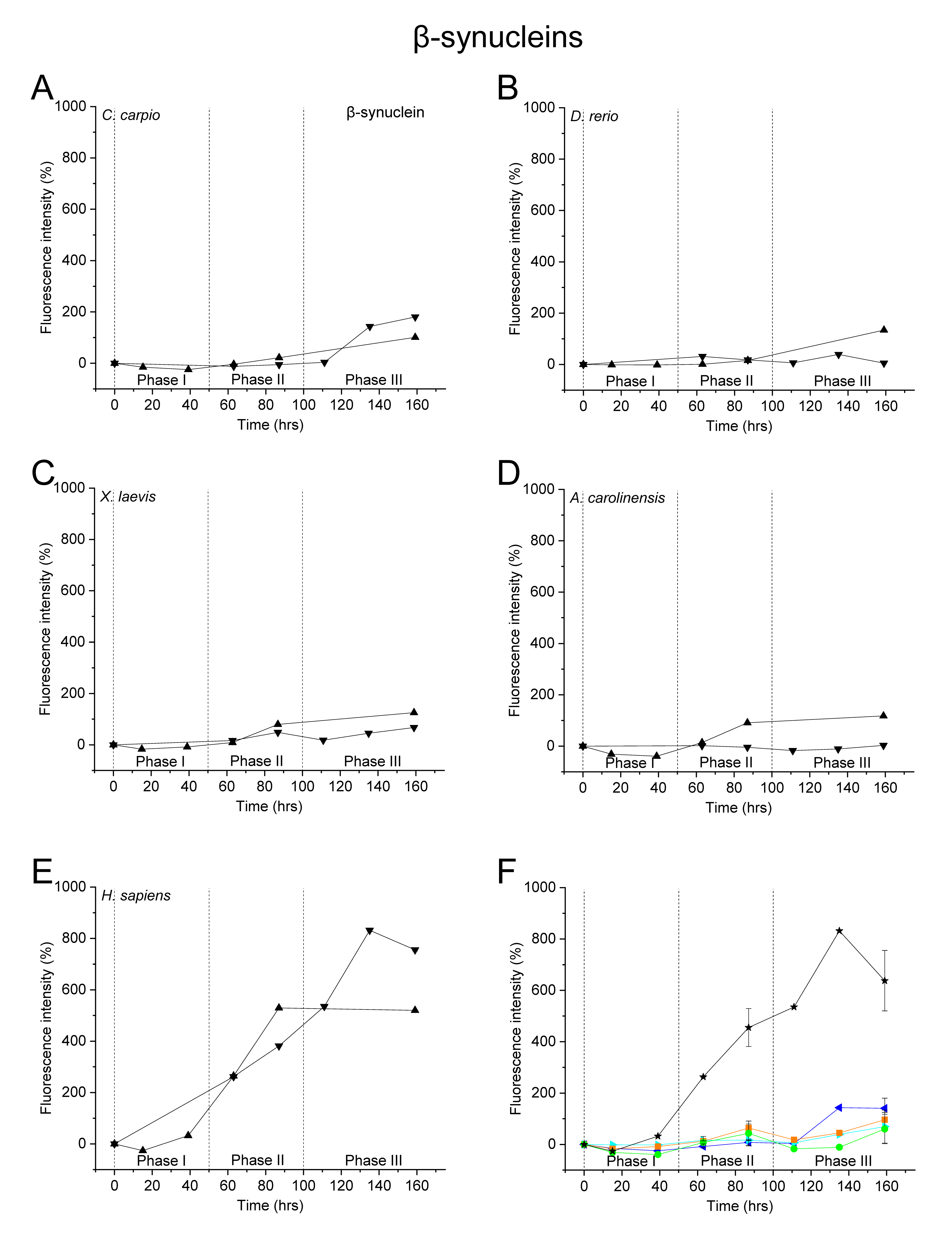

Supplement: Supplementary file 1 [file biomolecules-15-01231-s001.zip › Figure S8_600dpi (pixel-inch).tif]

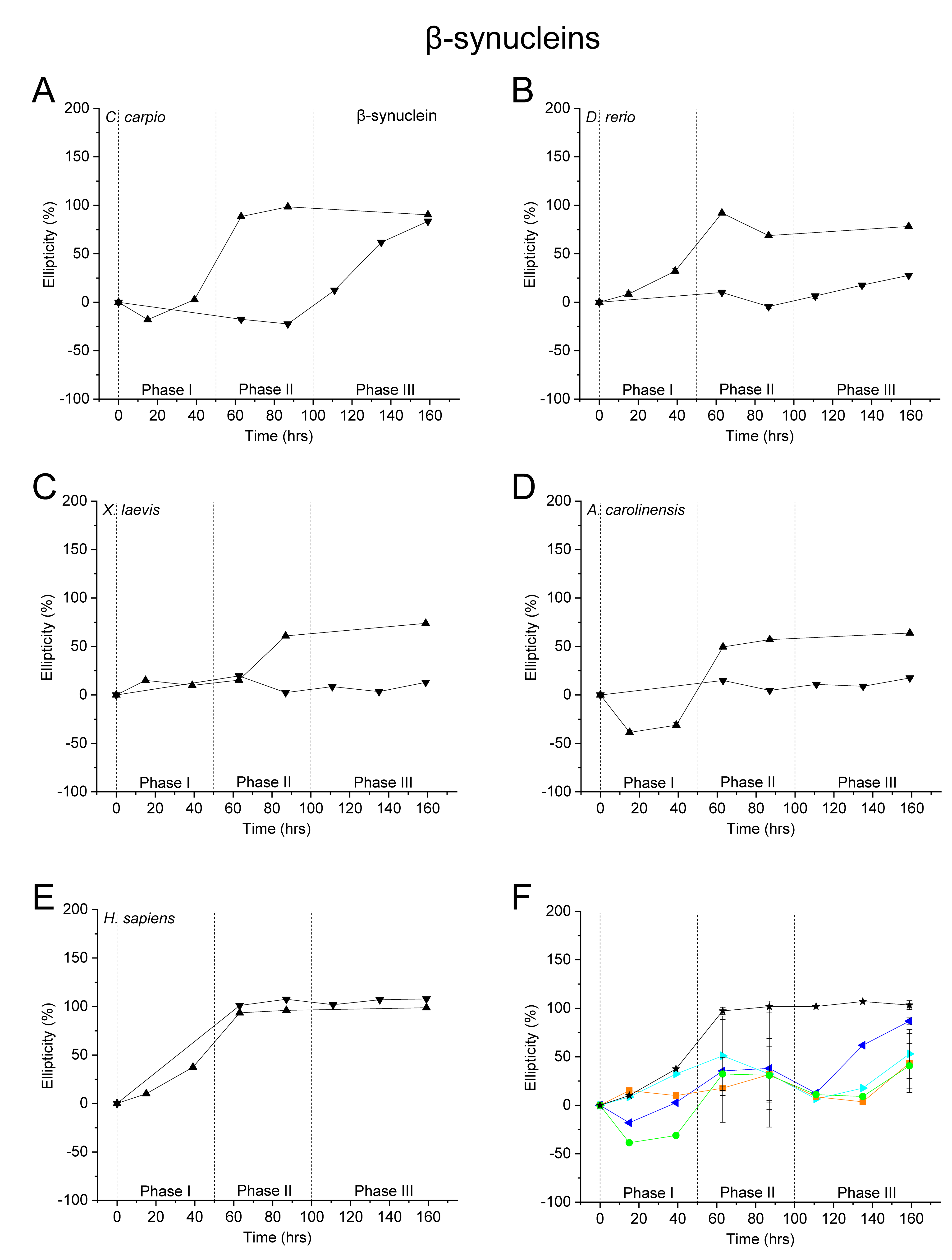

Supplement: Supplementary file 1 [file biomolecules-15-01231-s001.zip › Figure S9_600dpi (pixel-inch).tif]

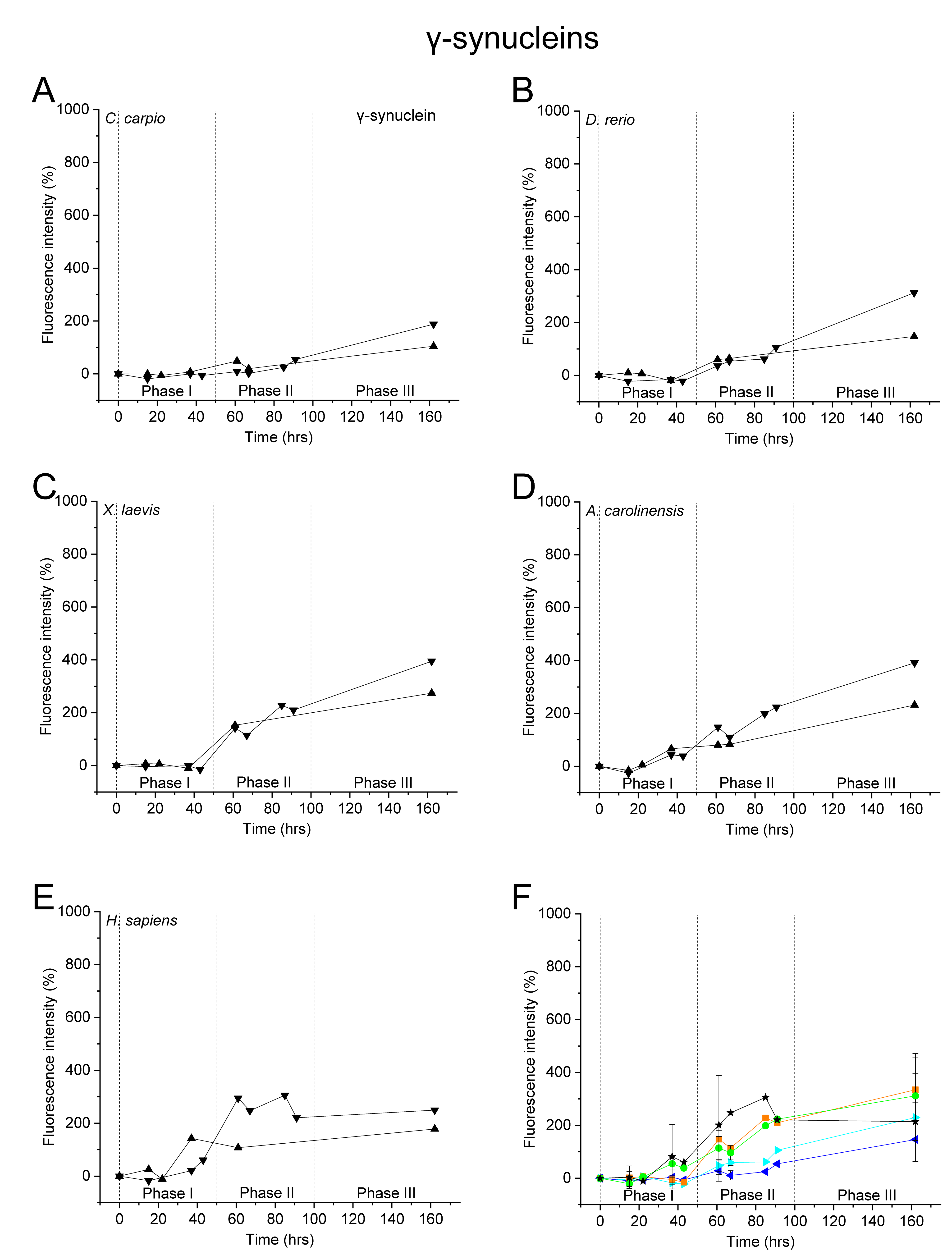

Supplement: Supplementary file 1 [file biomolecules-15-01231-s001.zip › Figure S10_600dpi (pixel-inch).tif]

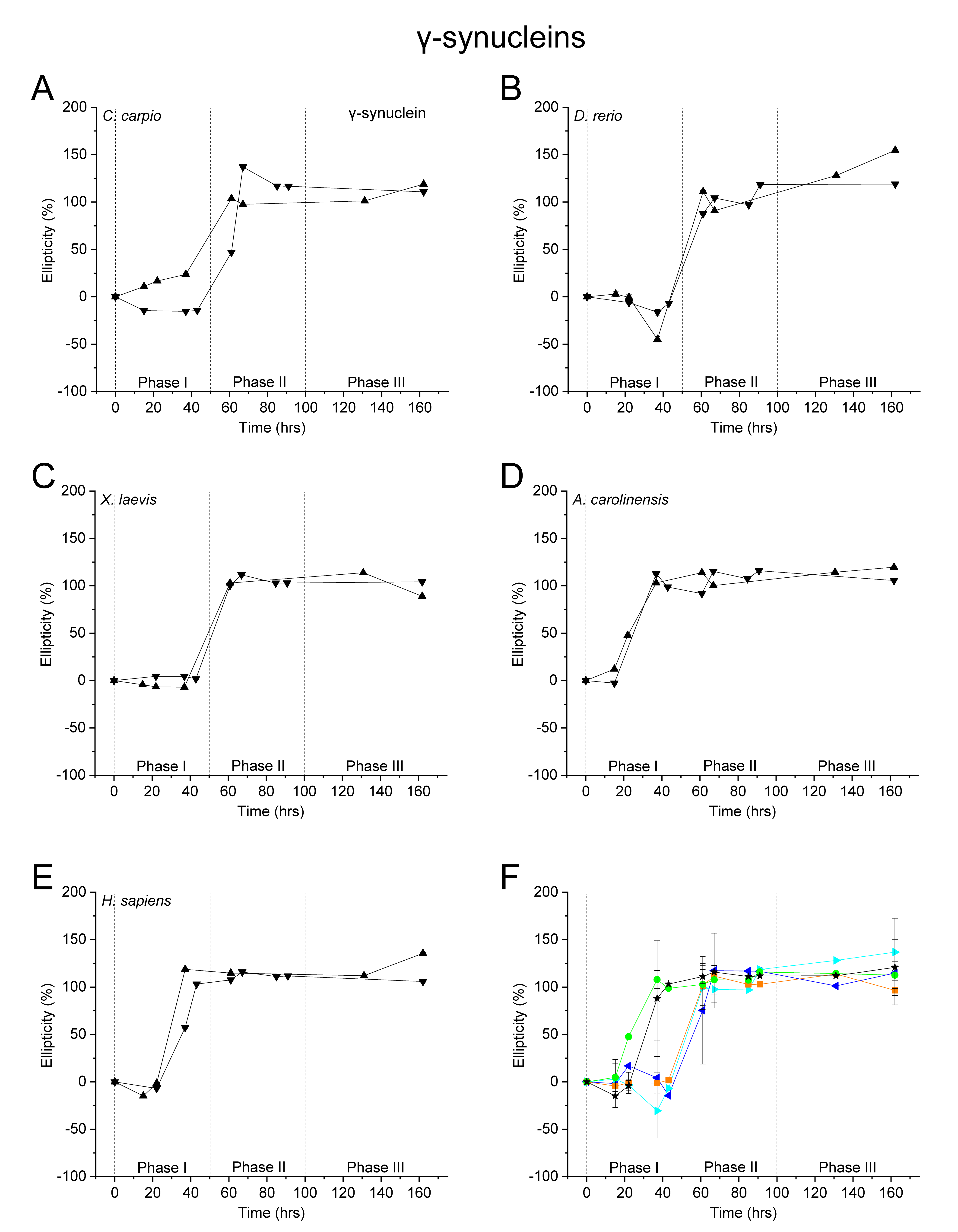

Supplement: Supplementary file 1 [file biomolecules-15-01231-s001.zip › Figure S11_600dpi (pixel-inch).tif]

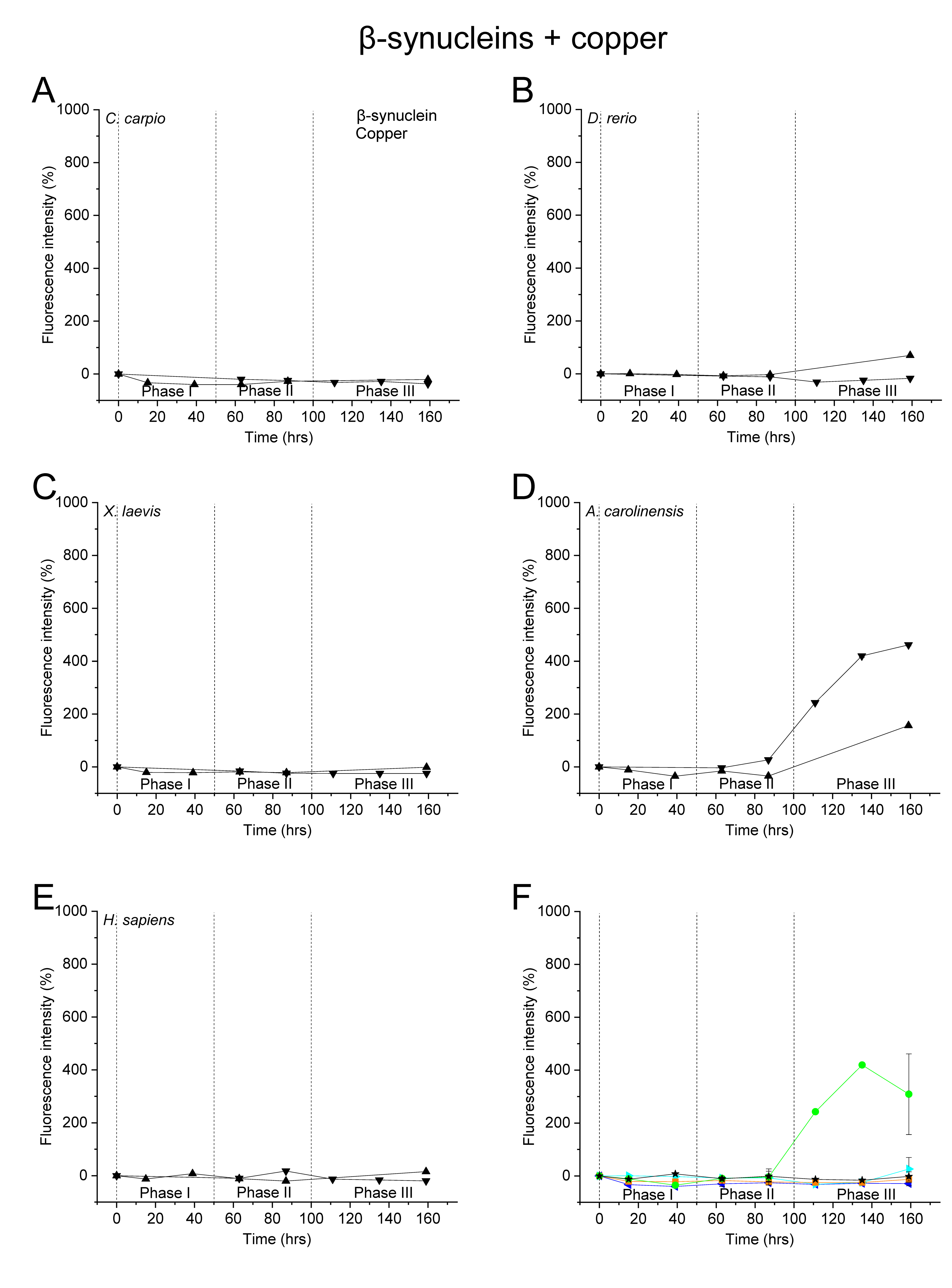

Supplement: Supplementary file 1 [file biomolecules-15-01231-s001.zip › Figure S12_600dpi (pixel-inch).tif]

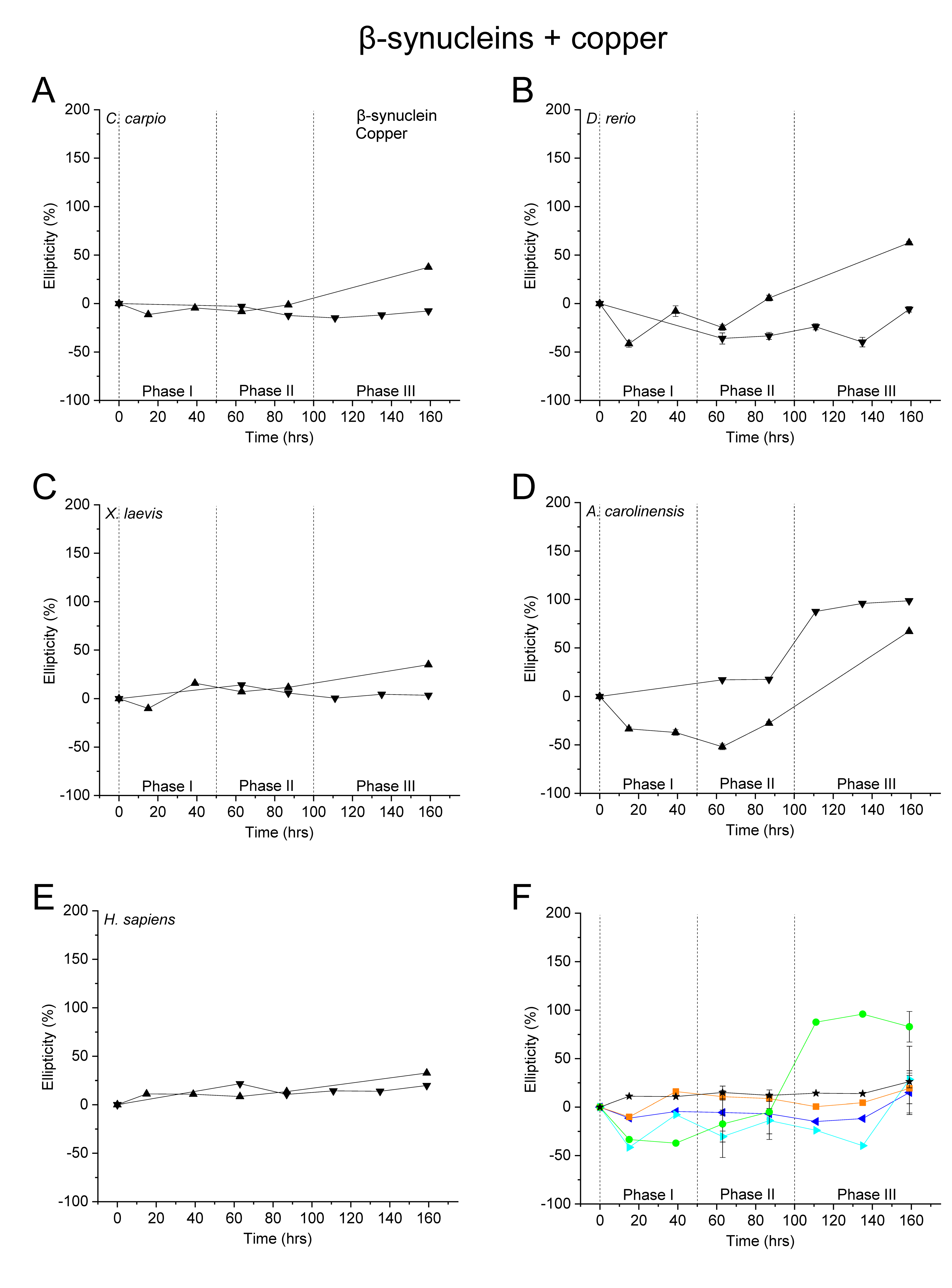

Supplement: Supplementary file 1 [file biomolecules-15-01231-s001.zip › Figure S13_600dpi (pixel-inch).tif]

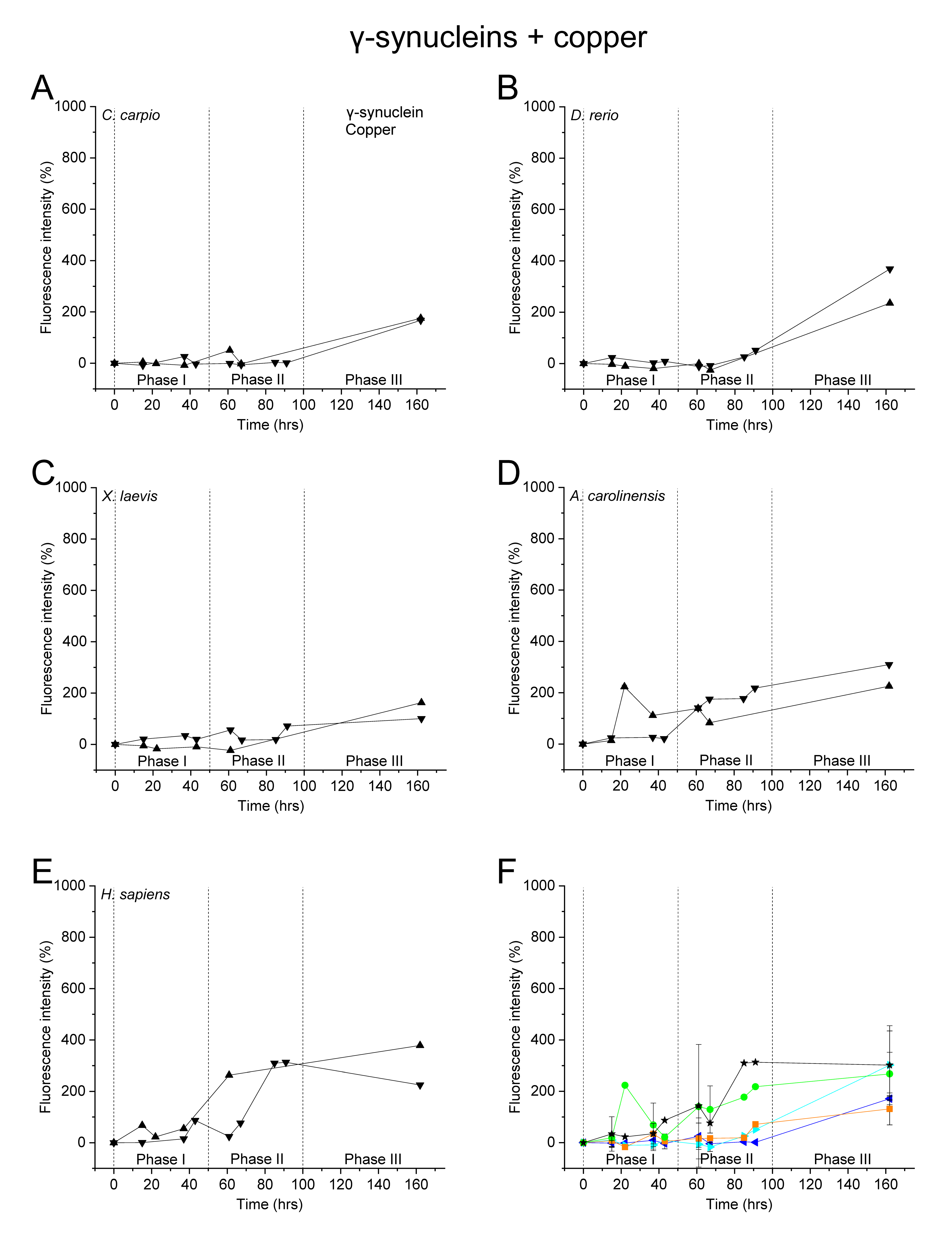

Supplement: Supplementary file 1 [file biomolecules-15-01231-s001.zip › Figure S14_600dpi (pixel-inch).tif]

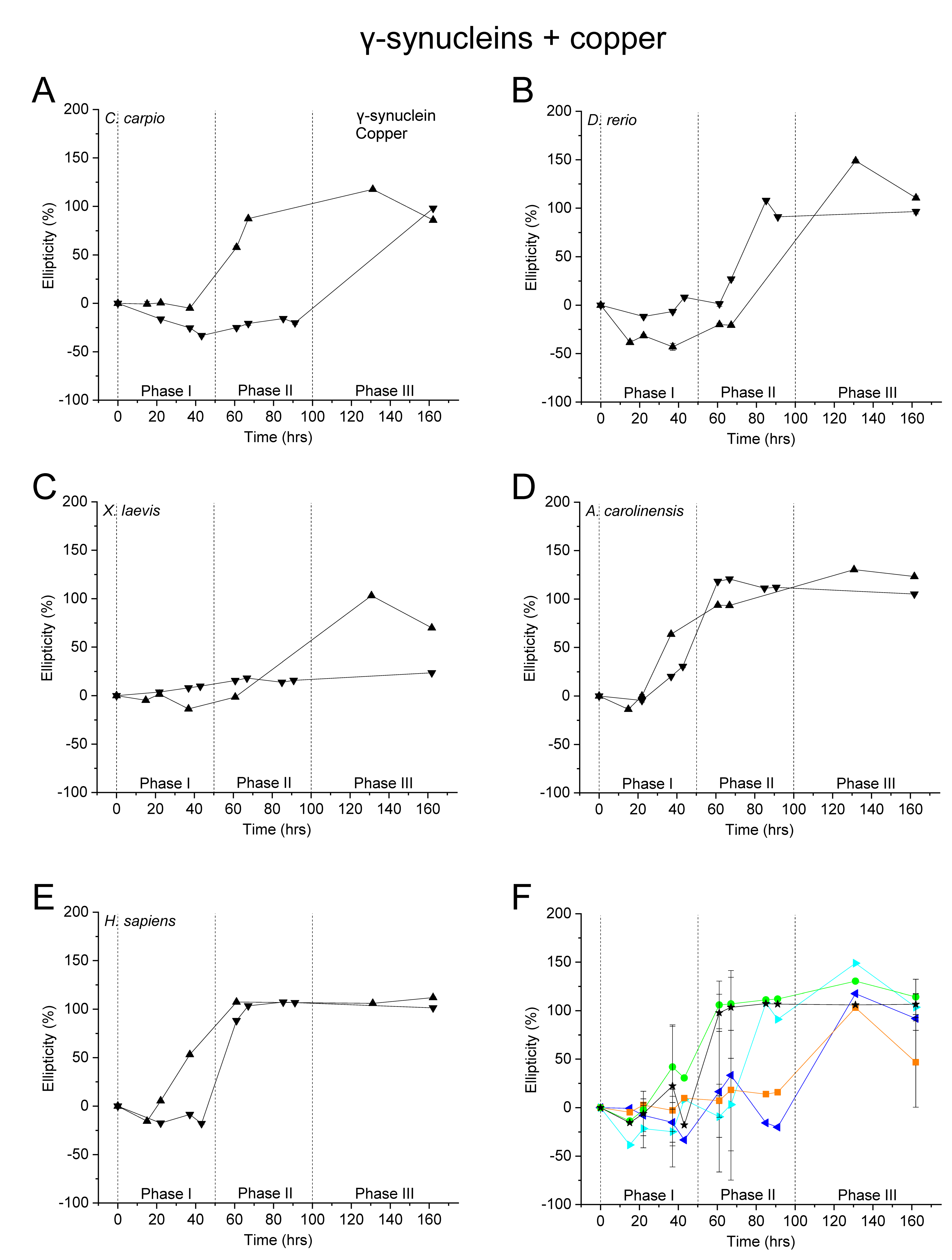

Supplement: Supplementary file 1 [file biomolecules-15-01231-s001.zip › Figure S15_600dpi (pixel-inch).tif]

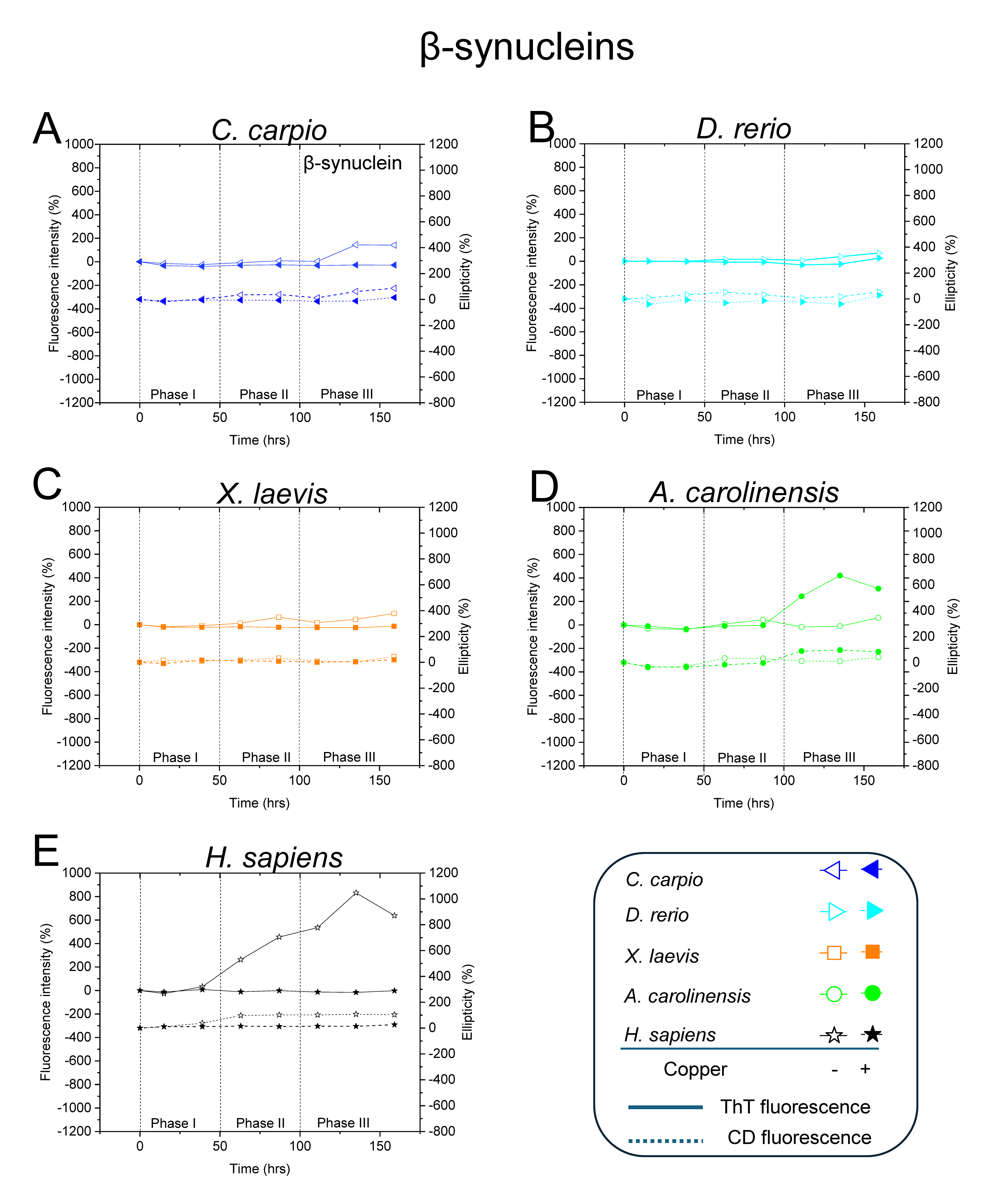

Supplement: Supplementary file 1 [file biomolecules-15-01231-s001.zip › Figure S16_600dpi (pixel-inch).tif]
